# Differential Effects of Liver Regeneration on Aging‐Related Changes in Gene Expression and Metabolic Function

**DOI:** 10.1111/acel.70197

**Published:** 2025-08-12

**Authors:** Ryo Murayama, Kenichi Horisawa, Shizuka Miura, Sumiaki Taniguchi, Jinghao Shu, Masatomo Takahashi, Yoshihiro Izumi, Takeshi Bamba, Kousei Ishigami, Atsushi Suzuki

**Affiliations:** ^1^ Division of Organogenesis and Regeneration Medical Institute of Bioregulation, Kyushu University Fukuoka Japan; ^2^ Department of Clinical Radiology, Graduate School of Medical Sciences Kyushu University Fukuoka Japan; ^3^ Division of Stem Cell Medicine Medical Institute of Bioregulation, Kyushu University Fukuoka Japan; ^4^ Division of Metabolomics Medical Institute of Bioregulation, Kyushu University Fukuoka Japan

**Keywords:** aging, gene expression, liver, metabolism, regeneration

## Abstract

Aging causes significant changes in gene expression and metabolic function of cells in various organs. Although it is known that liver regeneration is delayed by aging, the effects of aging on changes in gene expression and metabolic functions in liver regeneration need further investigation. In this study, we comprehensively analyzed changes in gene expression and metabolic function by liver regeneration in young and old mice to examine the effects of aging on these changes. During the process of liver regeneration, the gene expression profiles of hepatocytes from young and old mice changed significantly in a stepwise manner while each remained close together. After the completion of liver regeneration, the genes with aging‐specific expression patterns in old mouse hepatocytes changed to expression levels close to those in young mouse hepatocytes. In contrast to the results of these transcriptome analyses, the aging‐specific changes in metabolic state detected in old mouse livers were found to be largely maintained after the completion of liver regeneration. These results demonstrated that the gene expression state in the liver of old mice is flexibly altered by liver regeneration, whereas their metabolic state is robust. This finding helps to elucidate the relationship between aging and liver regeneration and to determine the basis of the increased incidence of liver disease with aging.

## Graphical abstract

The age‐specific gene expression status of aged mouse hepatocytes is mostly resolved and rejuvenated when liver regeneration is completed; whereas the age‐specific intracellular metabolic status detected in the liver of aged mice is largely maintained and not rejuvenated even after liver regeneration is completed.

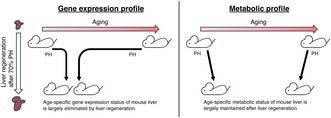

## 1 Introduction

Recent studies regarding aging in mammals have shown that gene expression changes in various organs have a significant impact on aging phenotypes (Fang et al. 2023; Garvey et al. 2014; Lu et al. 2004; Mori et al. 2007; Rizvi et al. 2021; Rupsingh et al. 2011; Zhang et al. 2021; Zheng et al. 2016). In the human cerebral cortex, the expression of genes involved in synaptic and mitochondrial functions decreases with age, resulting in a decline in cognitive function (Lu et al. 2004). In rat and human cardiomyocytes, aging alters the expression levels of genes related to the reactive oxygen species (ROS) homeostatic pathway and promotes increased ROS production, suggesting their involvement in aging‐related electrical and mechanical dysfunctions such as atrial fibrillation and heart failure (Rizvi et al. 2021). The liver is also affected by aging, and it has been reported that aging induces a decrease in the expression levels of genes encoding Cyp3a3, Cyp3a11, Cyp3a13, and Cyp3a18 in rat hepatocytes, resulting in a decrease in metabolic function due to aging (Mori et al. 2007).

Metabolic changes associated with aging occur in various organs and tissues, and many metabolic pathways are affected, including those involved in sugar and amino acid metabolism, and lipid and nucleic acid metabolism (Fang et al. 2023; Garvey et al. 2014; Rupsingh et al. 2011; Zhang et al. 2021; Zheng et al. 2016). Aging causes a decrease in fumaric acid, an intermediate of the tricarboxylic acid cycle, in the rat skeletal muscle and the mouse lung, suggesting that remodeling of energy metabolism occurs with aging (Garvey et al. 2014; Zhang et al. 2021). In the rat brain, the level of glutamate, which plays an important role as an excitatory neurotransmitter, decreases markedly with aging (Zheng et al. 2016). Glutamate is also significantly decreased in the brains of Alzheimer's disease patients (Rupsingh et al. 2011), suggesting a causal relationship between aging‐associated decreases in glutamate levels and the development of Alzheimer's disease.

The liver regulates many physiological processes including metabolism, bile formation and secretion, drug detoxification, glycogen storage, and protein synthesis. The liver has high regenerative capacity and can rapidly regain its mass after injury. Aging is known to reduce the regenerative capacity of the liver and delay the progression of liver regeneration (Bucher et al. 1964; Sánchez‐Hidalgo et al. 2012; Wang et al. 2001). Partial hepatectomy (PH), in which approximately 70% of the total liver mass is removed, is commonly used in studies of liver regeneration; it has been reported that the expression levels of several genes, including *Foxm1*, are altered by aging in the liver after PH, resulting in delayed liver regeneration (Wang et al. 2001). Interestingly, it has been reported that some genes whose expression levels are altered in the liver owing to aging return to the level of expression in the liver of young mice upon liver regeneration after PH, and that some of the characteristic patterns of H3K27me3 that arise in the liver during aging also revert to the state of young mice after liver regeneration (Chishti et al. 2013; Yang et al. 2023). These studies suggest that changes in gene expression and the epigenomic status of hepatocytes caused by aging are altered by liver regeneration, and that the aging state is eliminated. Although tissue rejuvenation by liver regeneration is intriguing, further studies are required to clarify the magnitude and time course of the effect of liver regeneration in alleviating the aging state of the liver at the gene expression level, and whether this effect ultimately affects changes in cellular metabolic function.

In this study, we isolated hepatocytes from the normal livers of young and old mice, as well as from the livers of mice during the process of liver regeneration after PH and after the completion of liver regeneration. We then comprehensively analyzed gene expression changes to understand the scale and temporal trends of the effects of liver regeneration in alleviating the aging condition of the liver at the gene expression level. In addition, by analyzing how the intracellular metabolic state in the livers of young and old mice changes after the completion of liver regeneration, we sought to determine whether the effect of liver regeneration on alleviating the aging state of the liver at the gene expression level extends to changes in cellular metabolic function.

## 2 Results

### 2.1 Age‐Dependent Differences in the Duration of Liver Regeneration in Mice

**FIGURE 1:**
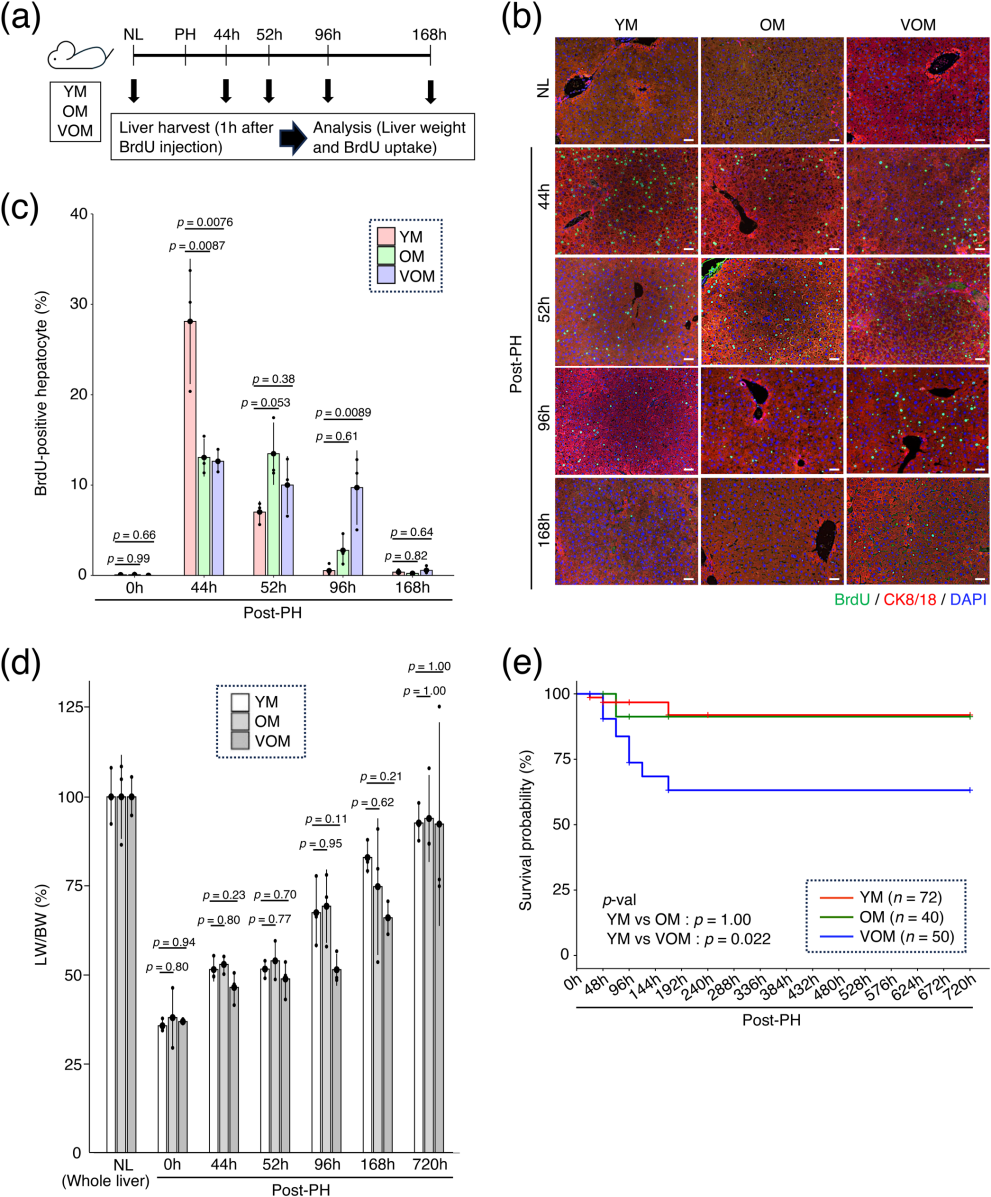
Delayed liver regeneration associated with aging. (a) Schematic diagram of experimental procedures. The weight and BrdU uptake of normal liver (NL) of YM, OM, and VOM and their regenerating livers at 44, 52, 96, and 168 h after PH were analyzed. Liver tissue was obtained from mice 1 h after intraperitoneal injection of BrdU. (b) Co‐immunofluorescence staining of BrdU with cytokeratin 8/18 (CK8/18) on the sections of NL and regenerating liver. DNA was stained with DAPI. Scale bars, 50 μm. (c) The graph shows the percentages of BrdU‐positive cells among CK8/18‐positive cells. Data represent means ± SD (*n* = 3 independent experiments) (d) Time course of the LW/BW ratio (%) measured immediately after PH (0 h) and at 44, 52, 96, 168, and 720 h after PH. Data represent means ± SD (*n* = 3 independent experiments) (e) Kaplan–Meier survival curves of indicated mice after PH. Statistical differences were determined by one‐way analysis of variance followed by Dunnett's test (c, d), and determined by Bonferroni's test (e).

**FIGURE 2:**
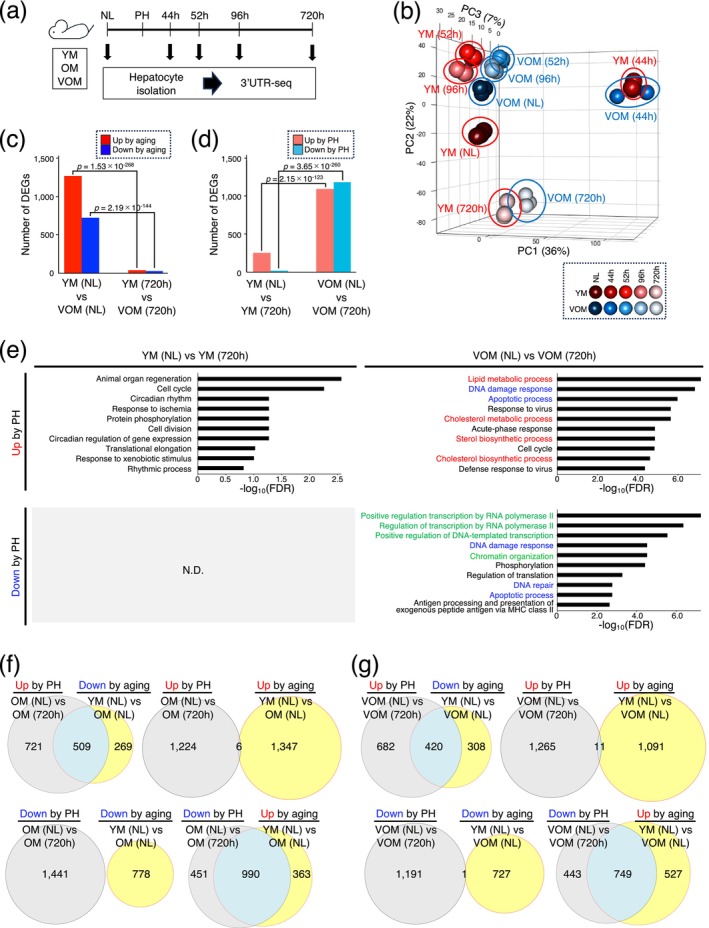
Dynamic changes in gene expression profiles of hepatocytes in VOM by liver regeneration. (a) Schematic diagram of experimental procedures. 3'UTR‐seq was conducted for hepatocytes isolated from normal liver (NL) of YM, OM, and VOM and their regenerating livers at 44, 52, 96, and 720 h after PH. At each analysis point, three mice each from YM, OM, and VOM groups were analyzed. (b) PCA was conducted for hepatocytes isolated from NL of YM and VOM and their regenerating livers using 3'UTR‐seq data. (c, d) The graphs show the number of DEGs identified by comparing gene expression profiles of hepatocytes isolated from NL of YM and VOM and from their livers at 720 h after PH, respectively (c), and those of hepatocytes isolated from NL of YM or VOM and their livers at 720 h after PH, respectively (d). (e) Top‐ten most significantly enriched GO terms associated with up‐ or down‐regulated genes in the livers of YM and VOM at 720 h after PH compared to NL of YM and VOM, respectively, identified by DAVID. GO terms associated with metabolic processes, apoptosis and DNA damage responses, and transcriptional regulation are highlighted in red, blue, and green, respectively. N.D., not detected. (f, g) Venn diagrams show direct comparisons of up‐ or down‐regulated genes between NL of OM or VOM and their livers at 720 h after PH, respectively, and those between NL of YM and that of OM or VOM, respectively. Statistical differences were determined by chi‐squared test (c, d), and determined by Fisher's exact test (e).

To evaluate the effect of aging on liver regeneration, we analyzed changes in the percentage of proliferating hepatocytes and liver weight over time after PH in 11–14 week‐old mice (young mice: YM), 60 week‐old mice (old mice: OM), and 100 week‐old mice (very old mice: VOM). The weights of the left and median lobes removed by PH and the weights of the right and caudate lobes left in the mouse after PH were measured to confirm that approximately 70% of the liver tissue was removed by PH (Figure S1). The uptake of 5‐bromo‐2′‐deoxyuridine (BrdU) in hepatocytes was analyzed at 44, 52, 96, and 168 h after PH (Figure 1a). As a result, the peak of BrdU uptake in hepatocytes of YM was detected 44 h after PH and decreased rapidly after 52 h (Figure 1b,c). In contrast, unlike YM, no peak in BrdU uptake was observed in the hepatocytes of the OM and VOM 44 h after PH, and BrdU uptake continued until 52 h after PH in the OM and until 96 h after PH in the VOM (Figure 1b,c). Because aging induces DNA damage, BrdU may potentially be incorporated into hepatocytes during DNA repair associated with such damage. Although a small number of cells expressing the phosphorylated form of H2A histone family member X (γH2AX), a biomarker of cellular response to DNA double‐strand breaks, were observed in the livers of VOM, these cells did not incorporate BrdU (Figure S2a,b). Moreover, transcriptome analysis during liver regeneration in YM, OM, and VOM (see Figure 2a) revealed that the timing of BrdU uptake in hepatocytes correlated with the expression of cell cycle‐related genes, but not with genes associated with the DNA damage responses (Figure S2c). Thus, it is suggested that, at least under the current BrdU labeling conditions (1 h), the likelihood of BrdU incorporation into hepatocytes during DNA repair is low.

When measuring the liver‐to‐body weight (LW/BW) ratio during liver regeneration, no statistically significant differences were observed between OM or VOM and YM due to large individual variations in body and liver weights in OM and VOM, given the group sizes used in this study. However, the timing of liver weight increase tended to be delayed in OM and VOM compared to YM (Figure 1d). The difference in the LW/BW ratio among the different age groups was observed even at 168 h post‐PH, suggesting that the process of liver regeneration was still ongoing, although the number of BrdU‐positive hepatocytes was significantly decreased. However, at 720 h after PH, the LW/BW ratio approached normal before PH in all age groups, suggesting that liver regeneration was complete at least 720 h after PH. The survival rates in YM and OM at 720 h after PH were 91.9% and 91.3%, respectively, whereas the survival rate in VOM was 63.1% (Figure 1e). It has been suggested that the lower survival rate of VOM than that of OM is due to a greater delay in liver regeneration. Taken together, these data confirm that liver regeneration after PH is delayed as mice age, as previously reported (Bucher et al. 1964; Sánchez‐Hidalgo et al. 2012; Wang et al. 2001). In addition, VOM appears to be more adversely affected by aging‐related impairments than OM, resulting in a prolonged delay in liver regeneration, which in turn reduces the survival rate of mice.

### 2.2 Elimination of Hepatocyte Gene Expression Patterns Specific to Aged Mice by Liver Regeneration

The gene expression changes that occur in hepatocytes during the liver regeneration process for YM, OM, and VOM were analyzed over time by 3′‐untranslated region sequencing (3'UTR‐seq) (Figure 2a). Principal component analysis (PCA) revealed that gene expression profiles changed significantly 44 h after PH, and subsequently tended to resemble those of hepatocytes in the OM and VOM at 52 and 96 h after PH (Figures 2b and S3a). At the completion of liver regeneration 720 h post‐PH, the gene expression profiles of hepatocytes in YM, OM, and VOM changed to a state different from their pre‐PH gene expression profiles (Figures 2b and S3a). Interestingly, YM and OM, and YM and VOM were found to have similar gene expression profiles at all time points of 44, 52, 96, and 720 h post‐PH, respectively (Figures 2b and S3a).

The number of differentially expressed genes (DEGs), extracted by comparing the gene expression profiles of hepatocytes isolated from YM and OM, and YM and VOM, was significantly reduced in livers at 720 h after PH compared to normal livers (Figures 2c and S3b). When comparing the gene expression profiles of hepatocytes isolated from normal livers of YM, OM, and VOM and those isolated from their livers at 720 h after PH, respectively, the number of DEGs identified in YM was significantly less than those identified in OM and VOM (Figures 2d and S3c). In the DEGs identified by comparing the gene expression profiles of hepatocytes isolated from YM normal livers and those from their livers at 720 h after PH, there were significantly fewer genes whose expression was decreased than those whose expression was increased (*p* = 1.11 × 10^−44^) (Figures 2d and S3c).

Gene ontology enrichment analysis (GOEA) was performed for the identified DEGs by comparing the gene expression profiles of hepatocytes isolated from normal livers of YM, OM, and VOM and those isolated from their livers at 720 h post‐PH. The results showed that the expression levels of genes related to animal organ regeneration, cell cycle, and circadian rhythm were elevated after PH in YM (Figure 2e), suggesting that a normal regenerative response occurred. In contrast, in OM and VOM, the expression levels of genes related to metabolism, such as lipid and cholesterol metabolic processes, apoptosis, and DNA damage responses, were upregulated after PH (Figures 2e and S3d). In addition, OM and VOM exhibited decreased expression of genes related to transcriptional regulation after PH, as well as decreased expression of a group of genes related to apoptosis and the DNA damage responses (Figures 2e and S3d). Many of the genes whose expression levels increased or decreased after PH in OM and VOM were genes whose expression levels were lower or higher in OM and VOM than in YM, respectively, in the DEGs identified by comparing hepatocytes isolated from normal livers of YM and OM, and YM and VOM (Figure 2f,g). To illustrate the interrelationships among the extracted genes, we presented the age‐related changes and post‐regeneration changes in expression levels of genes involved in cholesterol metabolism, steroid biosynthesis, and basal transcription factor pathways (Figure S4).

As both increased and decreased expression of genes associated with apoptosis and DNA damage responses were observed after PH in OM and VOM, we examined the genes in each GO term. We found that the expression of genes that positively drive apoptosis and DNA damage responses, including *Casp9*, *Apaf‐1*, *p21*, *Atm*, *Atr*, and *Topbp1*, was decreased after PH, whereas those that negatively drive them, including *Birc2*, *Pim2*, *Bcl6*, and *Bcl2l1*, increased after PH. Thus, it is suggested that apoptosis and DNA damage responses, which are known to be enhanced by aging (Duy et al. 2010; Fester et al. 2022; Fox et al. 2003; Harper and Elledge 2007; Kiraz et al. 2016; Schimmer 2004; Warren et al. 2019), are reduced after PH in OM and VOM hepatocytes.

These data demonstrate that the gene expression profiles of hepatocytes in OM and VOM change significantly after the completion of liver regeneration compared to hepatocytes of normal livers, and become relatively similar, but not identical, to the gene expression profiles of hepatocytes of normal livers of YM; therefore, hepatocyte gene expression patterns specific to aged mice are eliminated by liver regeneration.

### 2.3 Classification of Aging‐Specific Hepatocyte Gene Expression Patterns Eliminated by Liver Regeneration

**FIGURE 3:**
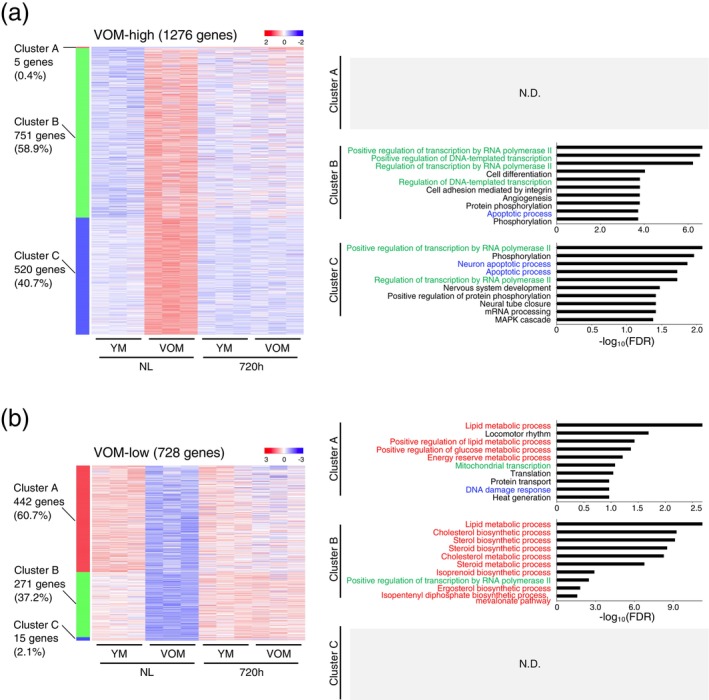
Changes in the expression of aging‐specific genes after completion of liver regeneration. (a, b) Heatmap images show changes at 720 h after PH in the expression of aging‐specific genes identified by comparing the gene expression profiles of hepatocytes isolated from normal liver (NL) of YM and VOM. For genes with higher (a) and lower (b) expression in NL of VOM compared to that of YM, they were classified into three clusters, respectively. Graphs at right depict the top‐ten most significantly enriched GO terms associated with genes in each cluster, as identified by DAVID. GO terms associated with metabolic processes, apoptosis and DNA damage responses, and transcriptional regulation are highlighted in red, blue, and green, respectively. N.D., not detected. Statistical differences were determined by Fisher's exact test.

**FIGURE 4:**
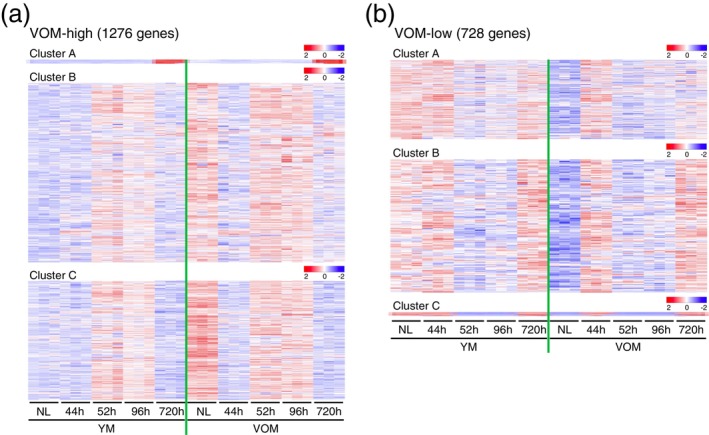
Sequential changes of aging‐specific gene expression during liver regeneration. (a, b) Heatmap images show sequential expression changes of aging‐specific genes classified into three clusters (see Figure 3), which were expressed at higher (a) or lower (b) levels in hepatocytes isolated from normal liver (NL) of VOM than those isolated from NL of YM. h, Hours.

As described above, the gene expression profiles of hepatocytes in YM, OM, and VOM 720 h after PH transitioned to a state relatively close to that of hepatocytes in normal YM livers, but not exactly the same. Therefore, we next classified the identified aging‐specific genes into two categories: genes whose expression levels in the hepatocytes of OM and VOM at 720 h after PH were relatively similar to those in the hepatocytes of YM normal livers, and genes whose expression levels were different. Among the DEGs identified through comparative analysis of gene expression profiles between hepatocytes from the normal livers of YM and those from OM or VOM, 48.3% and 40.7% of the genes were highly expressed in the hepatocytes of OM and VOM, respectively, and 16.5% and 37.2% of the genes were less expressed in the hepatocytes of OM and VOM, respectively, shifting to expression levels relatively close to those of hepatocytes in normal livers of YM at 720 h after PH (Figures 3, S5a,b, and S6). In contrast, 50.3% and 58.9% of the genes were highly expressed in the hepatocytes of OM and VOM, respectively, and 81.7% and 60.7% of the genes were less expressed in the hepatocytes of OM and VOM, respectively, transitioning to somewhat different expression levels from those in the hepatocytes of normal YM livers at 720 h after PH (Figures 3, S5a,b, and S6). The gene expression state at 720 h after PH in each of the classified clusters was already observed at 44 h after PH, and a temporary return to a gene expression state similar to that of hepatocytes in normal livers of OM and VOM was observed 52 and 96 h after PH in the livers of YM, OM, and VOM (Figures 4 and S5c,d).

Next, GOEA was performed on genes in each classified cluster. Among the genes that were more highly expressed in hepatocytes of normal OM and VOM livers than in those of YM normal livers, the expression levels of genes associated with transcriptional regulation, apoptosis, and DNA damage responses in the hepatocytes of YM, OM, and VOM at 720 h after PH were relatively similar to those in the hepatocytes of YM normal livers. In contrast, genes that showed partially different expression levels in hepatocytes of YM, OM, and VOM at 720 h after PH compared to those in normal livers of YM were mainly related to transcriptional regulation (Figures 3a and S5a). When focusing on genes that were less expressed in hepatocytes of OM and VOM normal livers than in those of YM normal livers, it was found that in hepatocytes of YM, OM, and VOM at 720 h after PH, there were genes whose expressions were increased to levels relatively similar to those of YM normal livers, and genes whose expression levels differed partially from those of YM normal livers, both of which contained a number of genes related to metabolic processes, including the lipid metabolic process (Figures 3b and S5b).

The expression levels of genes whose expression increased with aging, including many genes related to transcriptional regulation, either decreased to levels comparable to those of hepatocytes in normal YM livers at 720 h after PH; or their expression levels do not decrease to levels comparable to those of hepatocytes in normal YM livers. These results suggest that hepatocytes in YM, OM, and VOM at 720 h after PH may share a common abnormality involving transcriptional regulation. On the other hand, genes related to metabolic processes whose expression decreases in hepatocytes with aging also include genes that rose to levels comparable to those in hepatocytes of YM, OM, and VOM normal livers at 720 h after PH, and genes that failed to rise to that level, suggesting that certain metabolic processes are abnormal in hepatocytes at 720 h after PH.

### 2.4 Aging‐Associated Metabolic Changes in Aged Mouse Liver Are Maintained After Liver Regeneration

**FIGURE 5:**
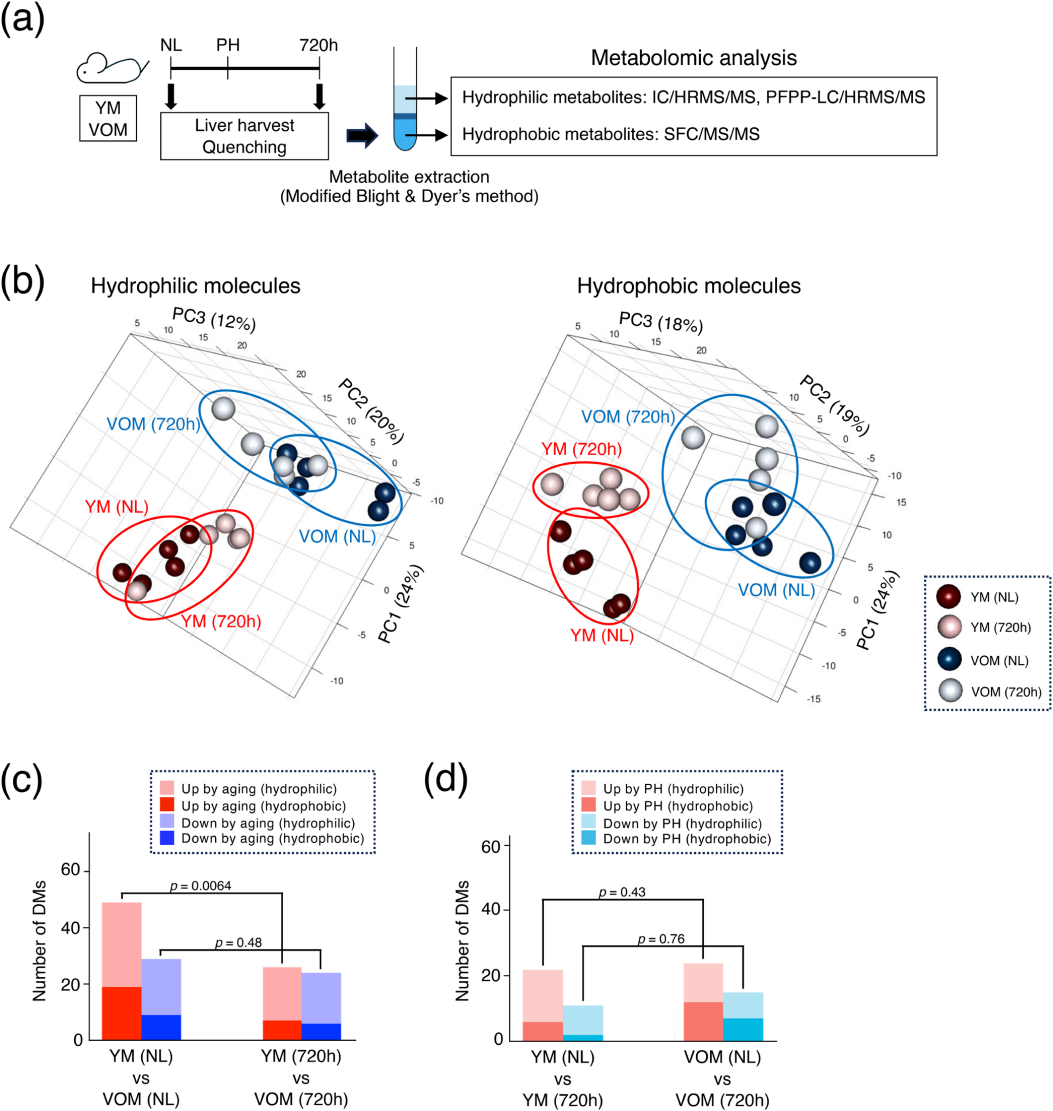
Metabolic changes that occur in aged mouse liver are maintained after liver regeneration. (a) Schematic diagram of experimental procedures. Metabolome analysis targeting both hydrophilic and hydrophobic metabolites was conducted for normal liver (NL) of YM and VOM and their livers at 720 h after PH. At each analysis point, five mice each from YM and VOM groups were analyzed. (b) PCA was conducted for NL of YM and VOM and their livers at 720 h after PH using the data of metabolome analysis. (c, d) The graphs show the number of DMs identified by comparing the metabolome profiles of NL of YM and VOM and from their livers at 720 h after PH, respectively (c), and those of NL of YM or VOM and their livers at 720 h after PH, respectively (d). Statistical differences were determined by chi‐squared test.

Transcriptome analysis performed in this study revealed that the gene expression profiles of hepatocytes in YM, OM, and VOM at 720 h after PH transitioned to a state close to that of hepatocytes in the normal livers of YM, suggesting that reprogramming of gene expression profiles occurred after the completion of liver regeneration. The expression of genes related to transcriptional regulation, apoptosis, and DNA damage responses, which increased with aging, decreased, whereas the expression of genes related to metabolic processes, which decreased with aging, increased. However, it is unclear whether these changes in gene expression levels actually lead to changes in the intracellular metabolic systems that control cellular functions and achieve metabolic reprogramming in the hepatocytes of OM and VOM at 720 h after PH. Thus, in this study, the normal livers of YM and VOM and their livers at 720 h after PH were collected for metabolomic analysis of hydrophilic and hydrophobic metabolites, respectively (Figure 5a).

In the PCA of metabolites, the profiles of hydrophilic and hydrophobic metabolites obtained from the normal livers of YM and VOM were very different, suggesting that the types of metabolites changed significantly with changes in cellular status due to aging (Figure 5b). In contrast, the profiles of hydrophilic and hydrophobic metabolites obtained from the livers of YM and VOM at 720 h after PH were similar to those of their respective normal livers. These data demonstrated that the types of metabolites altered by aging did not change significantly from those of the normal liver, even after liver regeneration was completed (Figure 5b).

**FIGURE 6:**
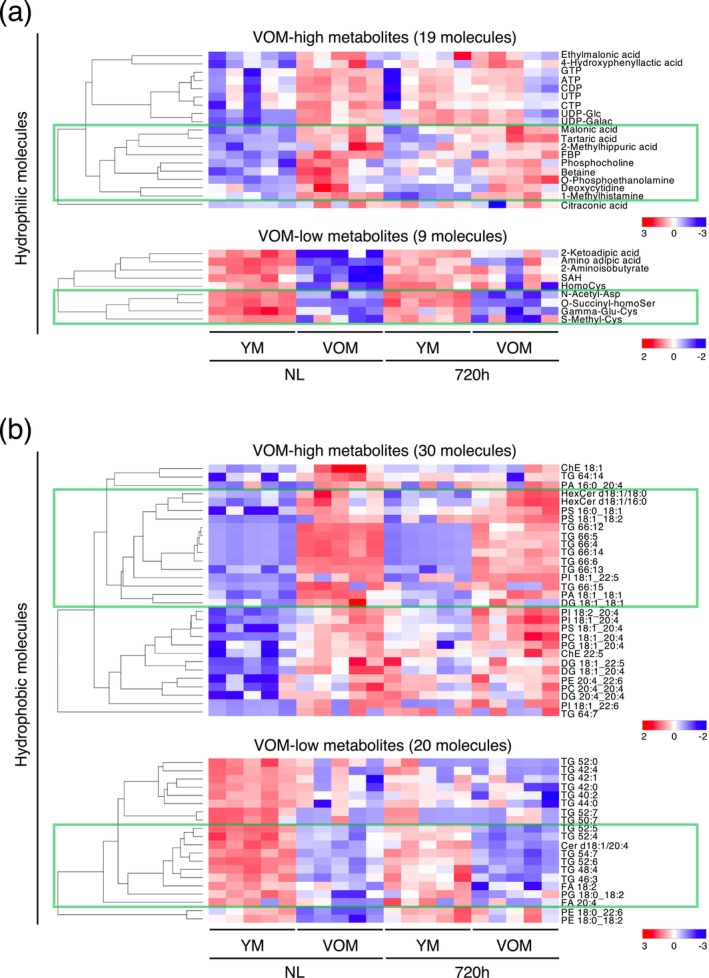
Many of the metabolites that are increased or decreased in aged mouse liver remain unchanged after liver regeneration. (a, b) Heatmap images show changes at 720 h after PH in the aging‐specific hydrophilic (a) and hydrophobic (b) metabolites identified by comparing the metabolome profiles of normal liver (NL) of YM and VOM. Metabolites with more or less in NL of VOM than in that of YM are shown separately. Metabolites indicated in green squares are DMs identified between NL of YM and VOM that were specifically maintained 720 h after PH.

The number of differential metabolites (DMs) identified by comparing the livers of YM and VOM revealed that the number of metabolites increased by aging was reduced to about half in the regenerated liver at 720 h after PH; whereas, the number of metabolites decreased by aging remained unchanged at 720 h after PH (Figure 5c). In contrast, the numbers of DMs that showed increased or decreased levels at 720 h after PH were similar between YM and VOM when compared to their respective normal livers (Figure 5d). Among the DMs that increased or decreased in the liver of VOM with aging, hydrophilic metabolites such as fructose 1,6‐bisphosphate (FBP), a metabolite involved in the rate‐limiting step of glycolysis, and betaine and phosphocholine, which are involved in the phospholipid metabolic pathway, as well as hydrophobic metabolites such as triglycerides (TG) and phospholipids of specific molecular weights, tended to be maintained even at 720 h after PH (Figure 6).

These data demonstrated that the changes in the intracellular metabolic systems that occur in the liver with aging are largely maintained after the completion of liver regeneration. Thus, although reprogramming of gene expression profiles is observed in the livers of aged mice after the completion of liver regeneration, reprogramming of the metabolic systems that control cellular functions does not occur. While the detailed mechanism remains unclear, one possible explanation is that the genes encoding proteins involved in intracellular metabolic pathways are subject to post‐transcriptional regulation, leading to changes in the abundance of these proteins. To examine this possibility, we focused on the phosphatidylcholine metabolic pathway, a representative lipid metabolism pathway, and analyzed age‐related and post‐regeneration changes in the expression levels of proteins associated with this pathway.

Metabolomic analysis revealed that the level of phosphocholine, an intermediate metabolite in the phosphatidylcholine metabolic pathway, was elevated in the liver of VOM and remained high 720 h after PH (Figure S7a). The abundance of phosphocholine is regulated by enzymes involved in its synthesis and metabolism, including choline kinase α (CHKA) and β (CHKB), which synthesize phosphocholine, and choline‐phosphate cytidylyltransferase (CCT) A (PCYT1A) and B (PCYT1B), which metabolize phosphocholine. Transcriptomic analysis showed that the expression of *Pcyt1a* in hepatocytes remained unchanged with aging or liver regeneration, and *Pcyt1b* expression was not detected. The expression of *Chka* increased with aging and decreased after liver regeneration, while *Chkb* expression was unaffected by either condition (Figure S7a). In contrast, western blotting analysis revealed that the protein levels of PCYT1A and CHKA decreased with aging and liver regeneration, whereas CHKB levels remained unchanged (Figure S7b,c). Although the reduction in CHKA could lead to decreased production of phosphocholine, the metabolic step catalyzed by CCT (PCYT1A and PCYT1B) is considered the rate‐limiting step in the pathway (Cornell and Ridgway 2015). Thus, the decrease in PCYT1A may have a more significant impact than the reduction of CHKA, potentially contributing to the observed increase in phosphocholine levels with aging and its maintenance after regeneration. These findings suggest that intracellular metabolism is tightly regulated by multiple proteins involved in complex metabolic pathways, and the abundance of these proteins can be controlled post‐transcriptionally. As a result, aging‐associated metabolic changes occurring in the liver may not be reset after the completion of liver regeneration, suggesting that the aging state of the liver is maintained.

## 3 Discussion

In this study, we performed PH in YM, OM, and VOM and analyzed changes in gene expression over time during subsequent liver regeneration. The manner of liver regeneration in young and old mice differed significantly, indicating that aging causes a delay in liver regeneration and the extent of this delay increases as aging progresses. Although the gene expression changes in hepatocytes caused by aging are remarkable, these age‐specific gene expression patterns quickly resolve in the early stages of liver regeneration after PH, regardless of the degree of aging. The gene expression profiles of hepatocytes in the livers of young and old mice changed significantly during liver regeneration, while they remained similar. Even after the completion of liver regeneration, hepatocytes in the livers of young and old mice exhibited similar gene expression profiles, which were relatively close to the gene expression profiles of hepatocytes in the normal livers of young mice. The majority of aging‐specific gene expression patterns were resolved after the completion of liver regeneration, suggesting that the metabolic state of the liver, which is altered by aging, may also exhibit a rejuvenating phenotype. However, the aging‐specific intracellular metabolic states detected in the livers of old mice did not necessarily lead to a rejuvenated metabolic state, even though the expression levels of genes associated with these metabolic pathways approached those in the hepatocytes of young mice.

Among the genes with aging‐specific expression patterns in the hepatocytes of old mice, there were genes whose expression levels changed to levels similar to those of the hepatocytes of young mice after the completion of liver regeneration, and genes whose expression levels approached but did not reach the same levels as those of the hepatocytes of young mice. Genes that showed expression levels close to those of young mouse hepatocytes accounted for approximately 30% of the total, suggesting that incomplete reprogramming of the gene expression state may prevent the recovery of metabolic function after the completion of liver regeneration. Another possible reason is the effect of aging on metabolic enzyme activities and translation inhibition caused by abnormal RNA modifications, which are known to contribute to mRNA splicing, nuclear export, transcript stability, and translation efficiency (Schmucker 2005; Shi et al. 2020). Aberrant RNA modifications may be implicated in the pathogenesis of age‐related diseases associated with metabolic syndromes, including cardiovascular disease and type 2 diabetes (Matsumura et al. 2023; McMahon et al. 2021). Another possibility is an increase in mitochondrial stress caused by ROS owing to reduced mitochondrial function in the hepatocytes of old mice (Miwa et al. 2022). Compared to the hepatocytes of young mice, the hepatocytes of old mice and those of young mice in which oxidative stress is induced by hydrogen peroxide treatment are known to enhance glucose and cholesterol uptake, and the secondary ROS generated promote fat accumulation in hepatocytes, thereby affecting liver metabolism (Seo et al. 2019). Because of these various factors, even if age‐specific gene expression signatures of hepatocytes are eliminated by liver regeneration, intracellular metabolic functions that change with aging are not reset by liver regeneration, and the aging state of the liver may not be restored.

This study revealed that the gene expression state in the livers of aged mice is flexibly altered by liver regeneration, whereas the metabolic state is robust, indicating that the aging state of the liver is not eliminated by the induction of regeneration. This finding is important for understanding the relationship between aging and liver regeneration, and can be used to elucidate the causes of the observed increased incidence of liver diseases with aging.

## 4 Methods

### 4.1 Mice

Young (11–14 week‐old), old (60 week‐old), and very old (100 week‐old) C57BL/6 mice (CLEA Japan) were used. The mice were bred in an environment maintained at a temperature of 22°C ± 4°C and a relative humidity of 60%, under a 12 h light/dark cycle (light: 08:00–20:00, dark: 20:00–08:00). The Animal Experiment Committee of Kyushu University approved the experimental protocol. All animals were provided with appropriate care, and the study was conducted in compliance with facility guidelines.

### 4.2 PH

Mice were anesthetized using isoflurane (Wako). The body surfaces of the mice were disinfected with ethanol, and a midline abdominal incision was made to expose the liver. The left and median lobes of the liver were ligated using 4‐0 silk sutures and subsequently resected. After resection, the abdominal incision was closed with 4‐0 silk sutures using the knotting technique. All surgeries were performed between 08:00 and 12:00.

### 4.3 Isolation of Hepatocytes

Hepatocytes were isolated from mouse livers using a two‐step collagenase digestion procedure (Seglen 1979). The mice were anesthetized with isoflurane, and their body surfaces were disinfected with ethanol. Laparotomy was performed, and a 22G Surflo (Terumo) catheter was inserted into the inferior vena cava. Thoracotomy was then performed, and the superior vena cava was clamped with a clip. To facilitate perfusion, the portal vein was severed with scissors to allow the perfusion solution injected via the catheter to flow from the inferior vena cava through the liver to the portal vein. The liver was perfused with a pre‐perfusion solution (10 mM HEPES, 5 mM glucose, 137 mM NaCl, 5.4 mM KCl, 4.2 mM NaHCO_3_, 0.85 mM Na_2_HPO_4_, 0.5 mM NaH_2_PO_4_, 0.5 mM EGTA), followed by perfusion with a solution of collagenase (Wako) at a concentration of 500 mg/L in Hank's balanced salt solution. After the two‐step perfusion, the liver was excised using surgical scissors, carefully dissected from the surrounding tissues, and minced in a sterile petri dish on ice. The hepatocytes were suspended in Hanks' balanced salt solution, filtered twice through sterile gauze, and centrifuged at 50× *g* for 1 min at 4°C. Non‐parenchymal cell fractions and impurities were removed from the supernatant, and hepatocyte pellets were collected. The isolated hepatocytes were washed four times with Hank's balanced salt solution and re‐isolated by centrifugation under the same conditions.

### 4.4 Fluorescent Immunostaining

To evaluate the proliferative potential of mouse hepatocytes, BrdU (Nacalai Tesque) was administered intraperitoneally at 50 mg/kg body weight 1 h before liver tissue collection. Paraffin sections of liver tissues were prepared for immunofluorescence staining as described previously (Miura and Suzuki 2017). Briefly, collected liver tissues were fixed in zinc formalin (Polysciences), dehydrated with graded ethanol and xylene, embedded in paraffin, and sectioned. The sections were deparaffinized, hydrated, and subjected to antigen retrieval by microwave heating in 0.01 M citrate buffer (pH 6.0). After washing with phosphate‐buffered saline (PBS), the sections were blocked with Block Ace (DS Pharma Biomedical). They were then incubated with primary antibodies including mouse anti‐BrdU (1:200; Amersham), guinea pig anti‐CK8/18 (1:500; Progen), and rabbit anti‐γH2AX (phospho S139) (1:2000; Abcam) antibodies. Following PBS washes, Alexa Fluor 488‐conjugated anti‐mouse IgG (1:2000; Thermo Fisher Scientific), Alexa Fluor 555‐conjugated anti‐guinea pig IgG (1:1000; Thermo Fisher Scientific), and Alexa Fluor 555‐conjugated anti‐rabbit IgG (1:2000; Thermo Fisher Scientific) antibodies were used as secondary antibodies. DNA was stained with DAPI (Wako).

### 4.5 Transcriptomic Analysis

Total RNA was extracted from hepatocytes freshly isolated from mouse livers using ISOGEN II (Nippon Gene) and purified using the RNeasy MinElute Cleanup Kit (Qiagen). To perform 3'UTR‐seq, a CEL‐seq2 method, we prepared a 3'UTR‐seq library and carried out sequencing using previously described procedures (Hashimshony et al. 2016; Inada et al. 2020). CEL‐seq2 was originally developed as a single‐cell RNA‐seq method (Hashimshony et al. 2016), but it is also available as an RNA‐seq method for cell populations (Goya et al. 2022; Inada et al. 2020; Miura et al. 2024). Initially, mRNA was reverse‐transcribed into cDNA using a SuperScript II Double‐Stranded cDNA Synthesis Kit (Thermo Fisher Scientific). The primers included a T7 promoter sequence, unique molecular identifiers (UMIs), sample‐specific barcodes, and a dT_n_ sequence to capture the poly(A) tail of the mRNA. The resulting cDNA served as a template for in vitro transcription using a T7 promoter to produce antisense RNA. The RNA was purified using RNAClean XP beads (Beckman Coulter), fragmented, and used for random priming and reverse transcription with random hexamers containing Illumina 3 adapter sequences. Next, a next‐generation sequencing (NGS) library was prepared using PCR amplification. The NGS libraries were size‐selected and purified using AMPure XP beads (Beckman Coulter), quantified, and quality‐checked using a BioAnalyzer 2100 (Agilent Technologies) using a High Sensitivity DNA Analysis Kit (Agilent Technologies), and further validated using a NEBNext Library Quant Kit for Illumina (New England Biolabs). Sequencing was performed using the Illumina HiSeq 1500 or NovaSeq 6000 NGS platforms.

### 4.6 Transcriptome Data Analysis

Raw data (FASTQ files) were mapped to the mouse reference genome (Ensembl GRCm38) using the yanailab/celseq2 pipeline and converted to UMI counts for each gene (Grün et al. 2014; Hashimshony et al. 2016). Next, using a method described by Grün et al. (2014), we analyzed transcriptome counts with a binomial distribution. In this method, if the UMI count reaches its maximum value of 4096, the transcriptome count diverges to infinity and is impossible to calculate. Therefore, we set the UMI count to 4096 for at least one sample. Specifically, the genes *Fga*, *Mt‐Rnr2*, *Mt‐Nd4*, *Mt‐Cytb*, *Mup14*, *Mup20*, *Mup8*, *Mup13*, and *Mup15* were excluded from analysis. We then filtered out low‐expression genes and performed batch correction of transcriptome counts between datasets using ComBat‐Seq (Zhang et al. 2020). Normalization and PCA were performed for batch‐corrected transcriptome data using the default settings of iDEP (v.0.96) (Ge et al. 2018). 2D and 3D plots were generated using R (v.4.1.1) with the R packages ggplot2 (v.3.4.2) and rgl (v.1.1.3), respectively. DEGs were detected using the default settings in iDEP (v.0.96), and GOEA of DEGs was conducted using the Database for Annotation, Visualization, and Integrated Discovery (DAVID; v2023q4) (Huang et al. 2009; Sherman et al. 2022). Clustering of DEGs was performed using the k‐means clustering function in the R package stats (v.4.4.1). The number of DEGs, their expression levels, and GOEA results were visualized using ggplot2 (v.3.4.2). Venn diagrams of DEGs were created using the R package Eulerr (v.7.0.0). Gene expression changes associated with aging, or those observed before and after PH, were mapped to KEGG pathways using iDEP (v.0.96) with default settings (Ge et al. 2018; Kanehisa and Goto 2000; Kanehisa et al. 2021; Weijun and Cory 2013). The analysis utilized the KEGG pathway maps: “Cholesterol metabolism,” “Steroid biosynthesis,” and “Basal transcription factors”.

### 4.7 Metabolomic Analysis

After euthanasia by cervical dislocation, the liver was excised within 3 min, placed in a 3 mL freezing and crushing tube, quenched in liquid nitrogen, and stored at −80°C. Frozen liver tissue was crushed using a multi‐bead shocker MB3200 (Yasui Kikai). Metabolite extraction was performed using the Bligh and Dyer method (Bligh and Dyer 1959) with certain modifications. Briefly, 20 ± 2 mg of frozen and pulverized liver tissue was placed in a 2 mL microtube and mixed with 970 μL of methanol, 10 μL of internal standard (IS) solution A (Mouse SPLASH Lipidomix Mass Spec Standard; Avanti Polar Lipids) containing phosphatidylcholine (PC) 15:0/[^2^H_7_]18:1 (1.0 nmol), phosphatidylethanolamine (PE) 15:0/[^2^H_7_]18:1 (0.070 nmol), phosphatidylserine (PS) 15:0/[^2^H_7_]18:1 (0.20 nmol), phosphatidylglycerol (PG) 15:0/[^2^H_7_]18:1 (0.050 nmol), phosphatidylinositol (PI) 15:0/[^2^H_7_]18:1 (0.20 nmol), phosphatidic acid (PA) 15:0/[^2^H_7_]18:1 (0.10 nmol), lysophosphatidylcholine (LPC) [^2^H_7_]18:1 (0.45 nmol), lysophosphatidylethanolamine (LPE) [^2^H_7_]18:1 (0.020 nmol), cholesteryl ester (ChE) [^2^H_7_]18:1 (2.5 nmol), diacylglycerol (DG) 15:0/[^2^H_7_]18:1 (0.15 nmol), triacylglycerol (TG) 15:0/[^2^H_7_]18:1/15:0 (0.35 nmol), and sphingomyelin (SM) d18:1/[^2^H_9_]18:1 (0.20 nmol), IS solution B (Avanti Polar Lipids) containing ceramide (Cer) d[^2^H_7_]18:1/15:0 (0.10 nmol), hexosylceramide (HexCer) d[^2^H_5_]18:1/18:1 (0.10 nmol), free fatty acid (FA) [^13^C_16_]16:0 (0.10 nmol), monoacylglycerol (MG) [^2^H_7_]18:1 (1.0 nmol), and [^2^H_7_]cholesterol (3.0 nmol), and IS solution C containing 10‐camphorsulfonic acid (1.5 nmol). The mixture was then vortexed for 1 min and sonicated for 5 min. To precipitate the proteins, methanol extracts were incubated on ice for 5 min. After centrifugation at 16,000× *g* for 5 min at 4°C, the supernatant was collected, and protein concentrations in the pellet were determined using a Pierce BCA Protein Assay Kit (Thermo Fisher Scientific). To facilitate phase separation, 400 μL of chloroform and 320 μL of water were added to 400 μL of the supernatant, followed by centrifugation at 16,000× *g* for 5 min at 4°C. The aqueous (upper) phase was collected, vacuum‐dried, and stored at −80°C until hydrophilic metabolite analysis. The dried samples were reconstituted in 50 μL of water before analysis. The organic (lower) phase was dried under nitrogen gas and stored at −80°C until lipidomic analysis. Before lipidomic analysis, the dried sample was reconstituted in 100 μL of methanol/chloroform (1/1, *v*/*v*). Hydrophilic metabolites were analyzed using two platforms: (1) Anionic polar metabolites (i.e., organic acids, nucleotides etc.) were analyzed via ion chromatography (IC) using a Dionex ICS‐5000^+^ HPIC system (Thermo Fisher Scientific) with a Dionex IonPac AG11‐HC‐4 μm guard column (2 mm i.d. × 50 mm, 4 μm particle size, Thermo Fisher Scientific) and a Dionex IonPac AS11‐HC‐4 μm column (2 mm i.d. × 250 mm, 4 μm particle size, Thermo Fisher Scientific) coupled with a Q Exactive, high‐performance benchtop quadrupole Orbitrap high‐resolution tandem mass spectrometer (Thermo Fisher Scientific) [IC/high‐resolution mass spectrometry (HRMS)/mass spectrometry (MS)] (Fushimi et al. 2020). (2) Cationic polar metabolites (i.e., amino acids, bases, nucleosides, etc.) were analyzed via liquid chromatography (LC) (Nexera X2 UHPLC system, Shimadzu) with a Discovery HS F5 column (2.1 mm i.d. × 150 mm, 3 μm particle size; Merck) coupled with a Q Exactive instrument (pentafluorophenylpropyl‐LC/HRMS/MS) (Fushimi et al. 2020). Both platforms were controlled using LabSolutions v.5.99 SP2 (Shimadzu) and Xcalibur v.4.2.47 (Thermo Fisher Scientific). Hydrophobic metabolites (i.e., lipids, etc.) were analyzed via supercritical fluid chromatography using a Nexera UC system (Shimadzu) equipped with an ACQUITY UPC2 Torus diethylamine column (3.0 mm i.d. × 100 mm, 1.7 μm particle size; Waters) coupled with a triple quadrupole mass spectrometry (LCMS‐8060; Shimadzu) (supercritical fluid chromatography/MS/MS) in multiple reaction monitoring (MRM) mode (Takeda et al. 2018). The analytical platform for lipidomic analysis was controlled using LabSolutions version 5.99 SP2.

### 4.8 Metabolome Data Analysis

Hydrophilic metabolites were identified by comparing the retention time (RT), HRMS, and HRMS/MS spectra of the samples with those of authentic standards analyzed under identical conditions (Sumner et al. 2007). Quantitative analysis of hydrophilic metabolites was conducted by normalizing the peak areas relative to the IS (10‐camphorsulfonic acid) and correcting for the total protein content of each sample. Hydrophobic metabolites were identified based on the RT and specific MRM transitions for each molecule (Takeda et al. 2018). Absolute quantification of hydrophobic metabolites was performed by calculating the ratio of the peak area of the stable isotope‐labeled IS for each lipid class to the peak area of the analyte, with subsequent correction for total protein content. Data were processed using Multi‐ChromatoAnalysT v.1.3.4.0 (BeForce) and Cascade v.1.1 (Reifycs). The resulting data matrix of quantitative values for hydrophilic and hydrophobic metabolites was subjected to PCA using MetaboAnalyst 6.0 (Pang et al. 2024). Normalization was performed with the following settings: sample normalization as “Normalization by median,” data transformation as “Log transformation,” and data scaling as “Auto scaling.” Plots were generated using the R software packages rgl (v.0.99.16), ggplot2 (v.3.4.2), and gplot (v.3.1.3).

### 4.9 Western Blotting and Protein Quantification

To analyze protein expression, whole cell lysates were prepared as follows: around 10^6^ hepatocytes freshly isolated from mouse livers were lysed in RIPA buffer containing protease inhibitors, with passaging through a 25G needle on ice. After determining protein concentrations with a BCA Protein Assay Kit (Pierce), the cell lysates were denatured with 2× SDS loading buffer at 95°C for 5 min. Then, electrophoresis was performed using 10% (*w*/*v*) SDS‐polyacrylamide gels, and 7.5 μg of total protein per well was loaded. Proteins were transferred to a polyvinylidene difluoride membrane (GE Healthcare) using a Trans‐Blot Turbo transfer system (Bio‐Rad). Subsequently, the blots were probed with primary antibodies, including rabbit anti‐PCYT1A (1:1000; Bioworld), rabbit anti‐CHKA (1:1000; Bioworld), mouse anti‐CHKB (1:1000; Santa Cruz Biotechnology), and mouse anti‐β‐actin (1:10,000; Abcam; horseradish peroxidase [HRP]‐conjugated) antibodies, after blocking with 5% (*w*/*v*) skim milk (Difco) in Tris‐buffered saline containing 0.1% (*v*/*v*) Tween‐20 (Nacalai Tesque) (TBS‐T). After washing several times with TBS‐T, the membranes were incubated with HRP‐conjugated secondary antibodies (1:2000; Abcam) specific to the species of the primary antibodies, except for β‐actin. Detection was performed with ECL Prime Western Blotting Detection Reagent (GE Healthcare) and an ImageQuant 800 system (Cytiva). Images were acquired in 24‐bit TIFF format and imported into Fiji software for quantification. Band intensities of each protein were measured, and the resulting signal values were normalized to the corresponding β‐actin signal.

### 4.10 Statistics and Reproducibility

The statistical significance of BrdU uptake into hepatocytes and the LW/BW ratio was assessed using one‐way analysis of variance followed by Dunnett's test. Kaplan–Meier survival curves were analyzed using the Bonferroni method. Statistical differences in GOEA data and the number of DEGs and DMs were determined using Fisher's exact test and chi‐squared test, respectively. Statistical analysis for protein expression data was performed using one‐way ANOVA for multiple comparisons, followed by Tukey's honestly significant difference test as a post hoc analysis. A *p*‐value of < 0.05 was considered statistically significant. The tissue images presented in the Figures were obtained from at least three independent experiments, and representative images are shown.

## Author Contributions

R.M., K.H., S.M., S.T., J.S., M.T., Y.I., and T.B. performed the experiments, collected the data, and conducted data analyses. A.S. contributed to the conception, design, and overall project management. R.M., K.H., and A.S. wrote the paper.

## Conflicts of Interest

The authors declare no conflicts of interest.

## Supporting information


**Figure S1:** acel70197‐sup‐0001‐FigureS1.pdf.

## Data Availability

All datasets, including the processed and batch‐correlated count matrix data, have been deposited in the Gene Expression Omnibus (GEO) database under the accession number GEO: GSE282210. In this study, we used batch‐corrected data from the matrix with the following column names: YM samples: YM_Ctrl_1_mix7, YM_Ctrl_2_mix7, YM_Ctrl_3_mix7, YM_44h_1_mix24, YM_44h_2_mix24, YM_44h_3_mix26, YM_52h_1_mix14, YM_52h_2_mix18, YM_52h_3_mix18, YM_4d_1_mix15, YM_4d_2_mix21, YM_4d_3_mix21, YM_1m_1_mix25, YM_1m_2_mix25, and YM_1m_3_mix27. OM samples: OM_Ctrl_1_mix16, OM_Ctrl_2_mix16, OM_Ctrl_3_mix16, OM_44h_1_mix24, OM_44h_2_mix24, OM_44h_3_mix26, OM_52h_1_mix16, OM_52h_2_mix17, OM_52h_3_mix23, OM_4d_1_mix16, OM_4d_2_mix20, OM_4d_3_mix20, OM_1m_1_mix25, OM_1m_2_mix25, and OM_1m_3_mix27. VOM samples: VOM_Ctrl_1_mix8, VOM_Ctrl_2_mix8, VOM_Ctrl_3_mix8, VOM_44h_1_mix24, VOM_44h_2_mix24, VOM_44h_3_mix26, VOM_52h_1_mix9, VOM_52h_2_mix9, VOM_52h_3_mix22, VOM_4d_1_mix13, VOM_4d_2_mix13, VOM_4d_3_mix19, VOM_1m_1_mix25, VOM_1m_2_mix25, and VOM_1m_3_mix27. All other data supporting the results presented herein are available within the article and Supporting Information and from the corresponding author upon reasonable request.

## References

[acel70197-bib-0001] Bligh, E. G. , and W. J. Dyer . 1959. “A Rapid Method of Total Lipid Extraction and Purification.” Canadian Journal of Biochemistry and Physiology 37, no. 8: 911–917. 10.1139/o59-099. 13671378

[acel70197-bib-0002] Bucher, N. R. , M. N. Swaffield , and A. F. Ditroiaf . 1964. “The Influence of Age Upon the Incorporation of Thymidine‐2‐C14into the DNA of Regenerating Rat Liver.” Cancer Research 24: 509–512. 14147827

[acel70197-bib-0003] Chishti, M. A. , N. Kaya , A. B. Binbakheet , F. Al‐Mohanna , M. H. Goyns , and D. Colak . 2013. “Induction of Cell Proliferation in Old Rat Liver Can Reset Certain Gene Expression Levels Characteristic of Old Liver to Those Associated With Young Liver.” Age 35, no. 3: 719–732. 10.1007/s11357-012-9404-z. 22477361 PMC3636416

[acel70197-bib-0004] Cornell, R. B. , and N. D. Ridgway . 2015. “CTP:Phosphocholine Cytidylyltransferase: Function, Regulation, and Structure of an Amphitropic Enzyme Required for Membrane Biogenesis.” Progress in Lipid Research 59: 147–171. 10.1016/j.plipres.2015.07.001. 26165797

[acel70197-bib-0005] Duy, C. , J. J. Yu , R. Nahar , et al. 2010. “BCL6 Is Critical for the Development of a Diverse Primary B Cell Repertoire.” Journal of Experimental Medicine 207, no. 6: 1209–1221. 10.1084/jem.20091299. 20498019 PMC2882829

[acel70197-bib-0006] Fang, W. , S. Chen , X. Jin , S. Liu , X. Cao , and B. Liu . 2023. “Metabolomics in Aging Research: Aging Markers From Organs.” Frontiers in Cell and Developmental Biology 11: 1198794. 10.3389/fcell.2023.1198794. 37397261 PMC10313136

[acel70197-bib-0007] Fester, N. , E. Zielonka , J. Goldmann , et al. 2022. “Enhanced Pro‐Apoptosis Gene Signature Following the Activation of TAp63α in Oocytes Upon γ Irradiation.” Cell Death & Disease 13, no. 3: 204. 10.1038/s41419-022-04659-2. 35246516 PMC8897389

[acel70197-bib-0008] Fox, C. J. , P. S. Hammerman , R. M. Cinalli , S. R. Master , L. A. Chodosh , and C. B. Thompson . 2003. “The Serine/Threonine Kinase Pim‐2 Is a Transcriptionally Regulated Apoptotic Inhibitor.” Genes & Development 17, no. 15: 1841–1854. 10.1101/gad.1105003. 12869584 PMC196230

[acel70197-bib-0009] Fushimi, T. , Y. Izumi , M. Takahashi , K. Hata , Y. Murano , and T. Bamba . 2020. “Dynamic Metabolome Analysis Reveals the Metabolic Fate of Medium‐Chain Fatty Acids in AML12 Cells.” Journal of Agricultural and Food Chemistry 68, no. 43: 11997–12010. 10.1021/acs.jafc.0c04723. 33073987

[acel70197-bib-0010] Garvey, S. M. , J. E. Dugle , A. D. Kennedy , et al. 2014. “Metabolomic Profiling Reveals Severe Skeletal Muscle Group‐Specific Perturbations of Metabolism in Aged FBN Rats.” Biogerontology 15, no. 3: 217–232. 10.1007/s10522-014-9492-5. 24652515 PMC4019835

[acel70197-bib-0011] Ge, S. X. , E. W. Son , and R. Yao . 2018. “iDEP: An Integrated Web Application for Differential Expression and Pathway Analysis of RNA‐Seq Data.” BMC Bioinformatics 19, no. 1: 534. 10.1186/s12859-018-2486-6. 30567491 PMC6299935

[acel70197-bib-0012] Goya, T. , K. Horisawa , M. Udono , et al. 2022. “Direct Conversion of Human Endothelial Cells Into Liver Cancer‐Forming Cells Using Nonintegrative Episomal Vectors.” Hepatology Communications 6, no. 7: 1725–1740. 10.1002/hep4.1911. 35220676 PMC9234650

[acel70197-bib-0013] Grün, D. , L. Kester , and A. Van Oudenaarden . 2014. “Validation of Noise Models for Single‐Cell Transcriptomics.” Nature Methods 11, no. 6: 637–640. 10.1038/nmeth.2930. 24747814

[acel70197-bib-0014] Harper, J. W. , and S. J. Elledge . 2007. “The DNA Damage Response: Ten Years After.” Molecular Cell 28, no. 5: 739–745. 10.1016/j.molcel.2007.11.015. 18082599

[acel70197-bib-0015] Hashimshony, T. , N. Senderovich , G. Avital , et al. 2016. “CEL‐Seq2: Sensitive Highly‐Multiplexed Single‐Cell RNA‐Seq.” Genome Biology 17: 77. 10.1186/s13059-016-0938-8. 27121950 PMC4848782

[acel70197-bib-0016] Huang, D. W. , B. T. Sherman , and R. A. Lempicki . 2009. “Systematic and Integrative Analysis of Large Gene Lists Using DAVID Bioinformatics Resources.” Nature Protocols 4, no. 1: 44–57. 10.1038/nprot.2008.211. 19131956

[acel70197-bib-0017] Inada, H. , M. Udono , K. Matsuda‐Ito , et al. 2020. “Direct Reprogramming of Human Umbilical Vein‐ and Peripheral Blood‐Derived Endothelial Cells Into Hepatic Progenitor Cells.” Nature Communications 11, no. 1: 5292. 10.1038/s41467-020-19041-z. PMC7578104 33087715

[acel70197-bib-0018] Kanehisa, M. , M. Furumichi , Y. Sato , M. Ishiguro‐Watanabe , and M. Tanabe . 2021. “KEGG: Integrating Viruses and Cellular Organisms.” Nucleic Acids Research 49, no. D1: D545–D551. 10.1093/nar/gkaa970. 33125081 PMC7779016

[acel70197-bib-0019] Kanehisa, M. , and S. Goto . 2000. “KEGG: Kyoto Encyclopedia of Genes and Genomes.” Nucleic Acids Research 28, no. 1: 27–30. 10.1093/nar/28.1.27. 10592173 PMC102409

[acel70197-bib-0020] Kiraz, Y. , A. Adan , M. Kartal Yandim , and Y. Baran . 2016. “Major Apoptotic Mechanisms and Genes Involved in Apoptosis.” Tumour Biology 37, no. 7: 8471–8486. 10.1007/s13277-016-5035-9. 27059734

[acel70197-bib-0021] Lu, T. , Y. Pan , S. Y. Kao , et al. 2004. “Gene Regulation and DNA Damage in the Ageing Human Brain.” Nature 429, no. 6994: 883–891. 10.1038/nature02661. 15190254

[acel70197-bib-0022] Matsumura, Y. , F. Y. Wei , and J. Sakai . 2023. “Epitranscriptomics in Metabolic Disease.” Nature Metabolism 5, no. 3: 370–384. 10.1038/s42255-023-00764-4. 36959512

[acel70197-bib-0023] McMahon, M. , C. Forester , and R. Buffenstein . 2021. “Aging Through an Epitranscriptomic Lens.” Nature Aging 1, no. 4: 335–346. 10.1038/s43587-021-00058-y. 37117595

[acel70197-bib-0024] Miura, S. , K. Horisawa , T. Iwamori , et al. 2024. “Hepatocytes Differentiate Into Intestinal Epithelial Cells Through a Hybrid Epithelial/Mesenchymal Cell State in Culture.” Nature Communications 15, no. 1: 3940. 10.1038/s41467-024-47869-2. PMC11096382 38750036

[acel70197-bib-0025] Miura, S. , and A. Suzuki . 2017. “Generation of Mouse and Human Organoid‐Forming Intestinal Progenitor Cells by Direct Lineage Reprogramming.” Cell Stem Cell 21, no. 4: 456–471. 10.1016/j.stem.2017.08.020. 28943029

[acel70197-bib-0026] Miwa, S. , S. Kashyap , E. Chini , and T. von Zglinicki . 2022. “Mitochondrial Dysfunction in Cell Senescence and Aging.” Journal of Clinical Investigation 132, no. 13: e158447. 10.1172/JCI158447. 35775483 PMC9246372

[acel70197-bib-0027] Mori, K. , P. E. Blackshear , E. K. Lobenhofer , et al. 2007. “Hepatic Transcript Levels for Genes Coding for Enzymes Associated With Xenobiotic Metabolism Are Altered With Age.” Toxicologic Pathology 35, no. 2: 242–251. 10.1080/01926230601156286. 17366318

[acel70197-bib-0028] Pang, Z. , Y. Lu , G. Zhou , et al. 2024. “MetaboAnalyst 6.0: Towards a Unified Platform for Metabolomics Data Processing, Analysis and Interpretation.” Nucleic Acids Research 52, no. W1: W398–W406. 10.1093/nar/gkae253. 38587201 PMC11223798

[acel70197-bib-0029] Rizvi, F. , C. C. Preston , L. Emelyanova , et al. 2021. “Effects of Aging on Cardiac Oxidative Stress and Transcriptional Changes in Pathways of Reactive Oxygen Species Generation and Clearance.” Journal of the American Heart Association 10, no. 16: e019948. 10.1161/JAHA.120.019948. 34369184 PMC8475058

[acel70197-bib-0030] Rupsingh, R. , M. Borrie , M. Smith , J. L. Wells , and R. Bartha . 2011. “Reduced Hippocampal Glutamate in Alzheimer Disease.” Neurobiology of Aging 32, no. 5: 802–810. 10.1016/j.neurobiolaging.2009.05.002. 19501936

[acel70197-bib-0031] Sánchez‐Hidalgo, J. M. , A. Naranjo , R. Ciria , et al. 2012. “Impact of Age on Liver Regeneration Response to Injury After Partial Hepatectomy in a Rat Model.” Journal of Surgical Research 175, no. 1: e1–e9. 10.1016/j.jss.2011.11.1022. 22341343

[acel70197-bib-0032] Schimmer, A. D. 2004. “Inhibitor of Apoptosis Proteins: Translating Basic Knowledge Into Clinical Practice.” Cancer Research 64, no. 20: 7183–7190. 10.1158/0008-5472.CAN-04-1918. 15492230

[acel70197-bib-0033] Schmucker, D. L. 2005. “Age‐Related Changes in Liver Structure and Function: Implications for Disease?” Experimental Gerontology 40, no. 8–9: 650–659. 10.1016/j.exger.2005.06.009. 16102930

[acel70197-bib-0034] Seglen, P. O. 1979. “Hepatocyte Suspensions and Cultures as Tools in Experimental Carcinogenesis.” Journal of Toxicology and Environmental Health 5, no. 2–3: 551–560. 10.1080/15287397909529766. 224209

[acel70197-bib-0035] Seo, E. , H. Kang , H. Choi , W. Choi , and H. S. Jun . 2019. “Reactive Oxygen Species‐Induced Changes in Glucose and Lipid Metabolism Contribute to the Accumulation of Cholesterol in the Liver During Aging.” Aging Cell 18, no. 2: e12895. 10.1111/acel.12895. 30609251 PMC6413652

[acel70197-bib-0036] Sherman, B. T. , M. Hao , J. Qiu , et al. 2022. “DAVID: A Web Server for Functional Enrichment Analysis and Functional Annotation of Gene Lists (2021 Update).” Nucleic Acids Research 50, no. W1: W216–W221. 10.1093/nar/gkac194. 35325185 PMC9252805

[acel70197-bib-0037] Shi, H. , P. Chai , R. Jia , and X. Fan . 2020. “Novel Insight Into the Regulatory Roles of Diverse RNA Modifications: Re‐Defining the Bridge Between Transcription and Translation.” Molecular Cancer 19, no. 1: 78. 10.1186/s12943-020-01194-6. 32303268 PMC7164178

[acel70197-bib-0038] Sumner, L. W. , A. Amberg , D. Barrett , et al. 2007. “Proposed Minimum Reporting Standards for Chemical Analysis: Chemical Analysis Working Group (CAWG) Metabolomics Standards Initiative (MSI).” Metabolomics 3, no. 3: 211–221. 10.1007/s11306-007-0082-2. 24039616 PMC3772505

[acel70197-bib-0039] Takeda, H. , Y. Izumi , M. Takahashi , et al. 2018. “Widely‐Targeted Quantitative Lipidomics Method by Supercritical Fluid Chromatography Triple Quadrupole Mass Spectrometry.” Journal of Lipid Research 59, no. 7: 1283–1293. 10.1194/jlr.D083014. 29724780 PMC6027907

[acel70197-bib-0040] Wang, X. , E. Quail , N. J. Hung , Y. Tan , H. Ye , and R. H. Costa . 2001. “Increased Levels of Forkhead Box M1B Transcription Factor in Transgenic Mouse Hepatocytes Prevent Age‐Related Proliferation Defects in Regenerating Liver.” Proceedings of the National Academy of Sciences 98, no. 20: 11468–11473. 10.1073/pnas.201360898. PMC58753 11572993

[acel70197-bib-0041] Warren, C. F. A. , M. W. Wong‐Brown , and N. A. Bowden . 2019. “BCL‐2 Family Isoforms in Apoptosis and Cancer.” Cell Death & Disease 10, no. 3: 177. 10.1038/s41419-019-1407-6. 30792387 PMC6384907

[acel70197-bib-0042] Weijun, L. , and B. Cory . 2013. “Pathview: An R/Bioconductor Package for Pathway‐Based Data Integration and Visualization.” Bioinformatics 29, no. 14: 1830–1831. 10.1093/bioinformatics/btt285. 23740750 PMC3702256

[acel70197-bib-0043] Yang, N. , J. R. Occean , D. P. Melters , et al. 2023. “A Hyper‐Quiescent Chromatin State Formed During Aging Is Reversed by Regeneration.” Molecular Cell 83, no. 10: 1659–1676.e11. 10.1016/j.molcel.2023.04.005. 37116496 PMC10228348

[acel70197-bib-0044] Zhang, F. , J. Kerbl‐Knapp , A. Akhmetshina , et al. 2021. “Tissue‐Specific Landscape of Metabolic Dysregulation During Ageing.” Biomolecules 11, no. 2: 235. 10.3390/biom11020235. 33562384 PMC7914945

[acel70197-bib-0045] Zhang, Y. , G. Parmigiani , and W. E. Johnson . 2020. “ComBat‐Seq: Batch Effect Adjustment for RNA‐Seq Count Data.” NAR Genomics and Bioinformatics 2, no. 3: lqaa078. 10.1093/nargab/lqaa078. 33015620 PMC7518324

[acel70197-bib-0046] Zheng, X. , T. Chen , A. Zhao , et al. 2016. “The Brain Metabolome of Male Rats Across the Lifespan.” Scientific Reports 6: 24125. 10.1038/srep24125. 27063670 PMC4827083

