# Supplementary material for: Differential Effects of Liver Regeneration on Aging‐Related Changes in Gene Expression and Metabolic Function
Source: Aging Cell. 2025 Aug 12;24(10):e70197. doi: 10.1111/acel.70197 (PMC12507397; doi:10.1111/acel.70197)
Supplement: Supplementary file 1 — Figure S1: acel70197‐sup‐0001‐FigureS1.pdf. [file ACEL-24-e70197-s001.pdf]

SUPPLEMENTARY INFORMATION

Differential effects of liver regeneration on aging-related changes in gene expression and metabolic function

Ryo Murayama et al.

(a)

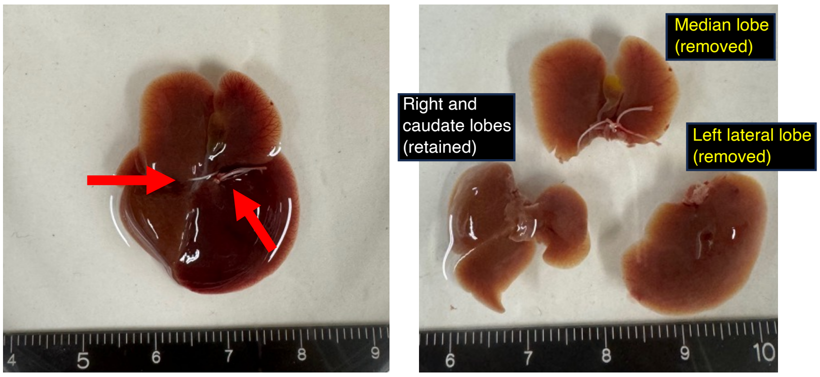

(b)

Sample	Left lateral lobe (g)	Median lobe (g)	Right and caudate lobes (g)	Resected liver tissue / Total liver tissue
1	0.37	0.35	0.35	67.3 %
2	0.31	0.35	0.26	71.7 %
3	0.49	0.44	0.49	65.5 %
**Mean ± SD**	0.39 ± 0.07	0.38 ± 0.04	0.37 ± 0.09	68.2 ± 2.6 %

Figure S1. Confirmation of the accuracy of our PH methodology.

(**a**) Representative images of the whole liver harvested immediately after ligation (left panel), left and median lobes of the liver removed by PH (right panel), and right and caudate lobes of the liver retained after PH (right panel). Red arrows indicate the thread used in PH. (**b**) The weights of the left and median lobes of the liver that were removed by PH and the right and caudate lobes of the liver that were retained after PH were measured, and the percentages of resected liver tissue to total liver tissue were calculated. Data represent means ± SD (*n* = 3 independent experiments).

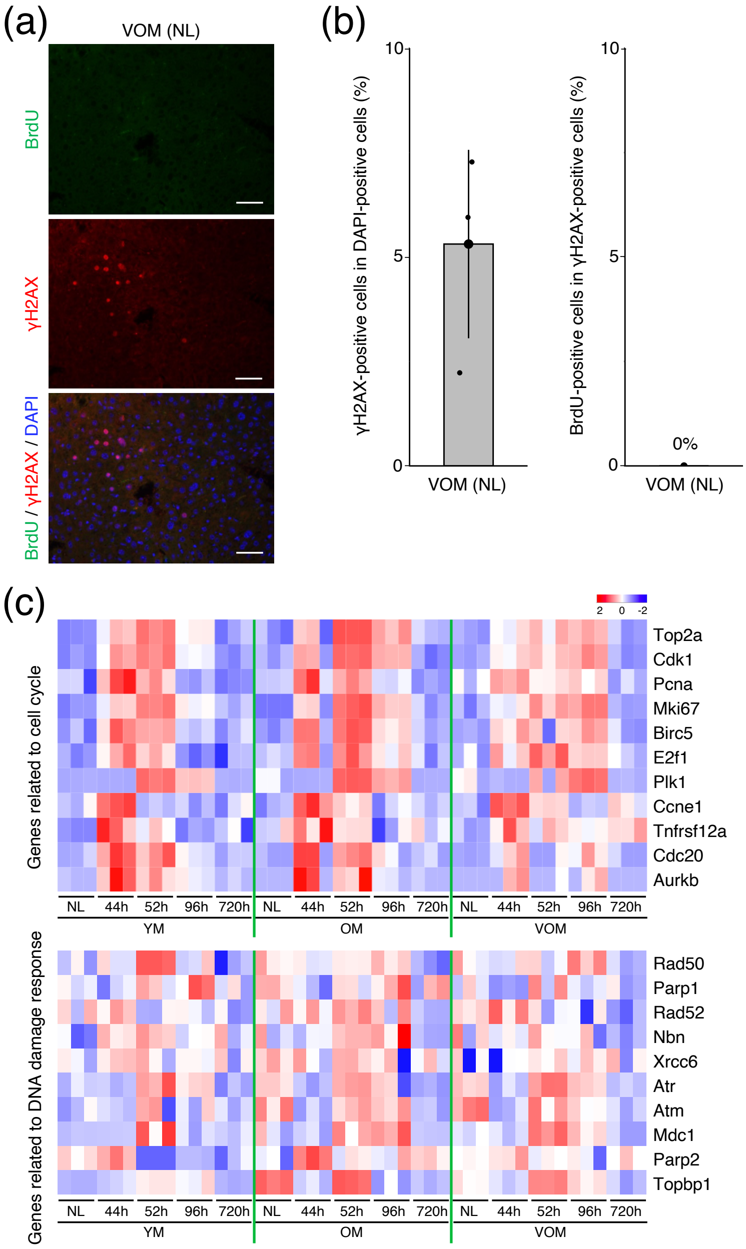

Figure S2. BrdU labeling primarily detects DNA synthesis rather than DNA repair. (**a**) Co-immunofluorescence staining of BrdU with γH2AX on the sections of normal liver (NL) of VOM. DNA was stained with D API. Scale bars, 50 μm. (**b**) The percentages of γH2AX-positive cells among DAPI-positive cells (left graph) and BrdU-positive cells among γH2AX-positive cells (right graph) in NL of VOM. (**c**) Heatmap images show sequential expression changes of genes associated with the cell cycle and DNA damage response in hepatocytes isolated from NL of YM, OM, and VOM and their regenerating livers at 44 hours (h), 52 h, 96 h, and 720 h after PH.

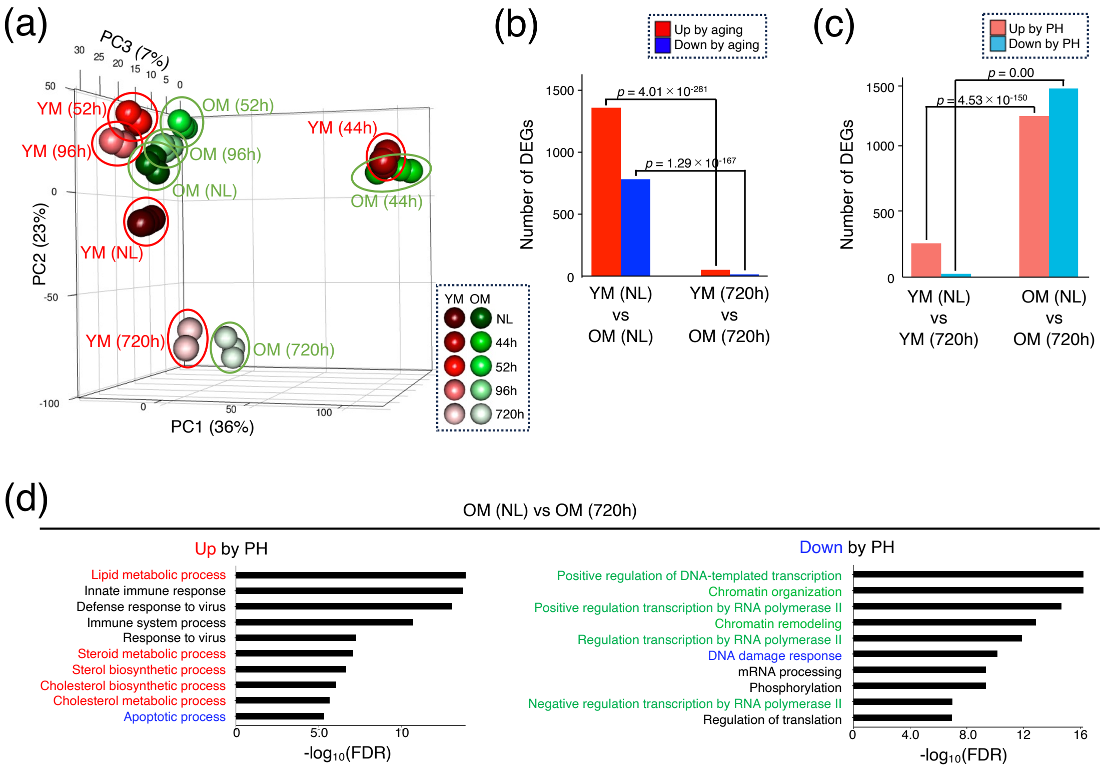

Figure S3. Dynamic changes in gene expression profiles of hepatocytes in OM by liver regeneration.

(**a**) PCA was conducted for hepatocytes isolated from normal liver (NL) of YM and OM and their regenerating livers at 44 hours (h), 52 h, 96 h, and 720 h after PH using 3'UTR-seq data. (**b**, **c**) The graphs show the number of DEGs identified by comparing the gene expression profiles of hepatocytes isolated from the NL of YM and OM and from their livers 720 h after PH (**b**), and those of hepatocytes isolated from the NL of YM or OM and their livers 720 h after PH (**c**). (**d**) Top-ten most significantly enriched GO terms associated with up- or downregulated genes in the livers of OM at 720 h after PH compared to NL of OM, respectively, as identified by DAVID. GO terms associated with metabolic processes, apoptosis and DNA damage responses, and transcriptional regulation are highlighted in red, blue, and green, respectively. Statistical differences were determined by Chi-square test (**b**, **c**), and determined by Fisher's exact test (**d**).

(a)

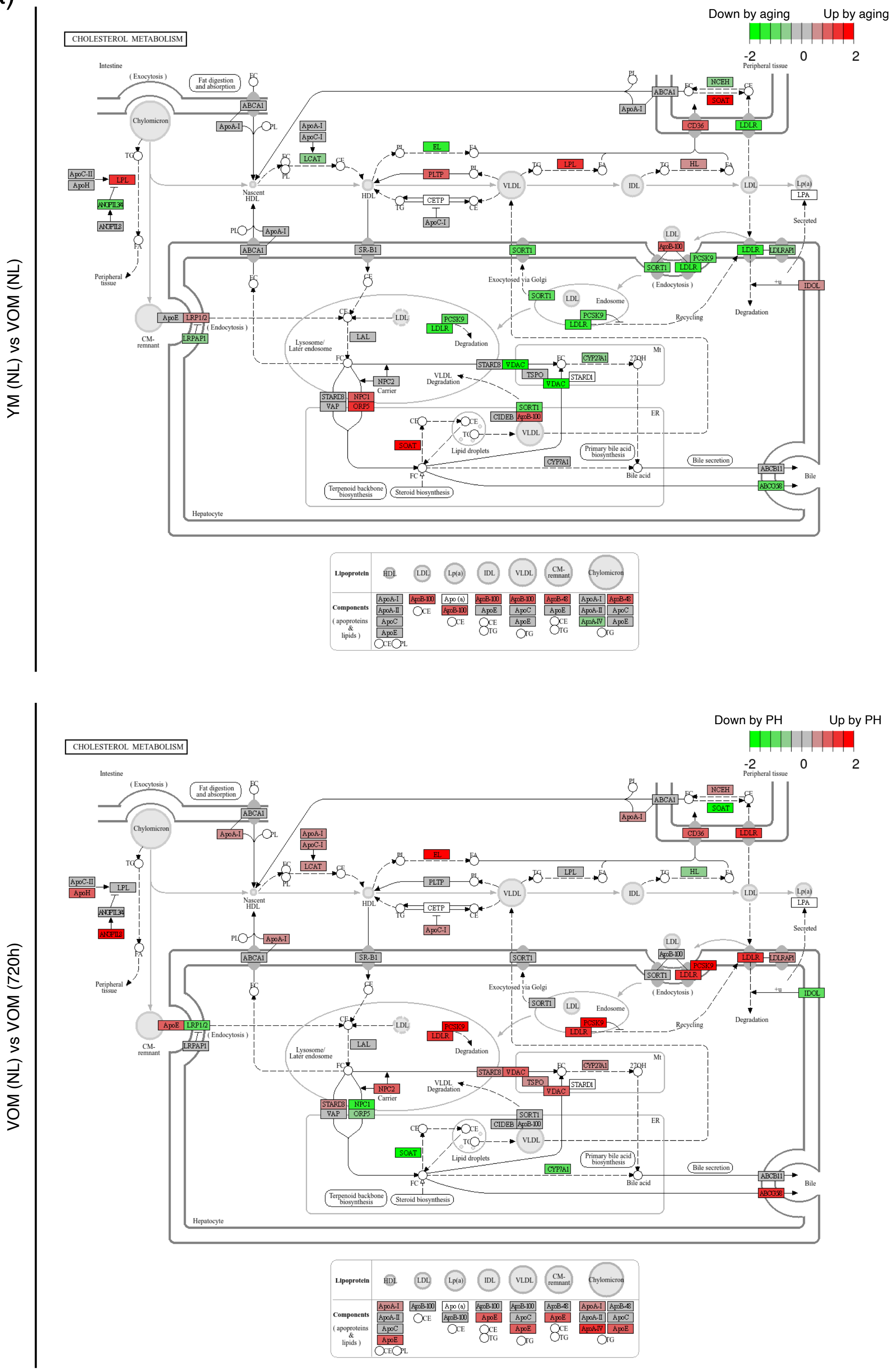
YM (NL) vs VOM (NL)
Down by aging
Up by aging
-2
0
2
CHOLESTEROL METABOLISM
VOM (NL) vs VOM (720h)
Down by PH
Up by PH
-2
0
2
CHOLESTEROL METABOLISM

(b)

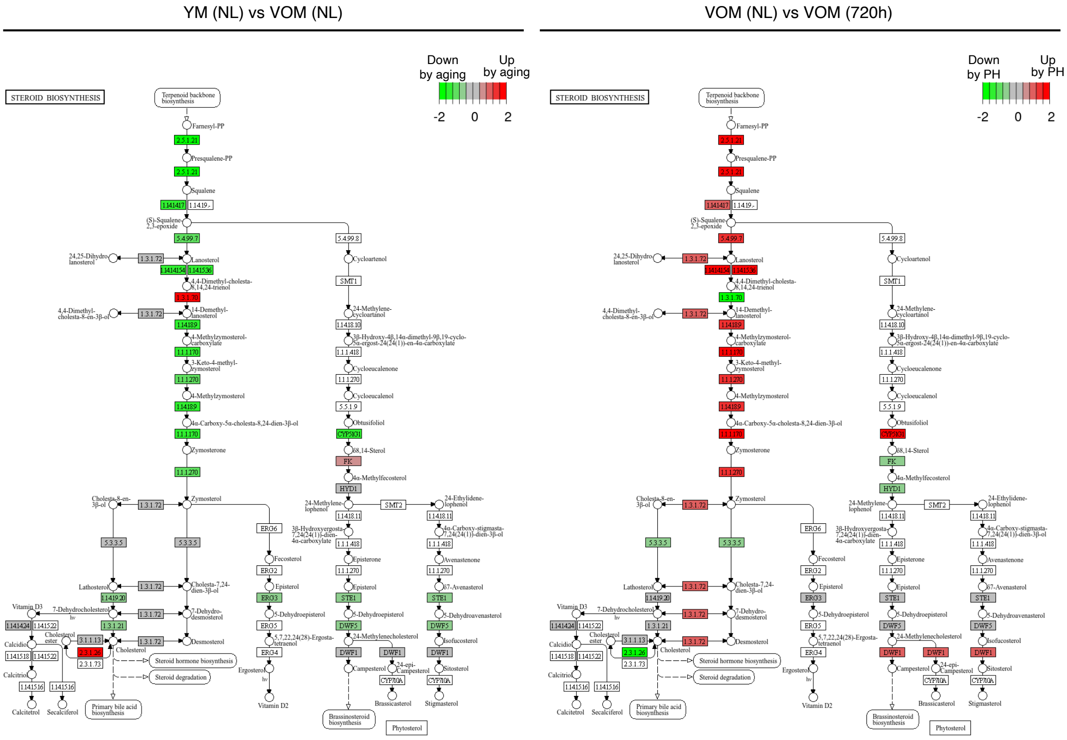
YM (NL) vs VOM (NL)
VOM (NL) vs VOM (720h)
Down by aging
Up by aging
-2 0 2
Down by PH
Up by PH
-2 0 2
STEROID BIOSYNTHESIS
STEROID BIOSYNTHESIS

(c)

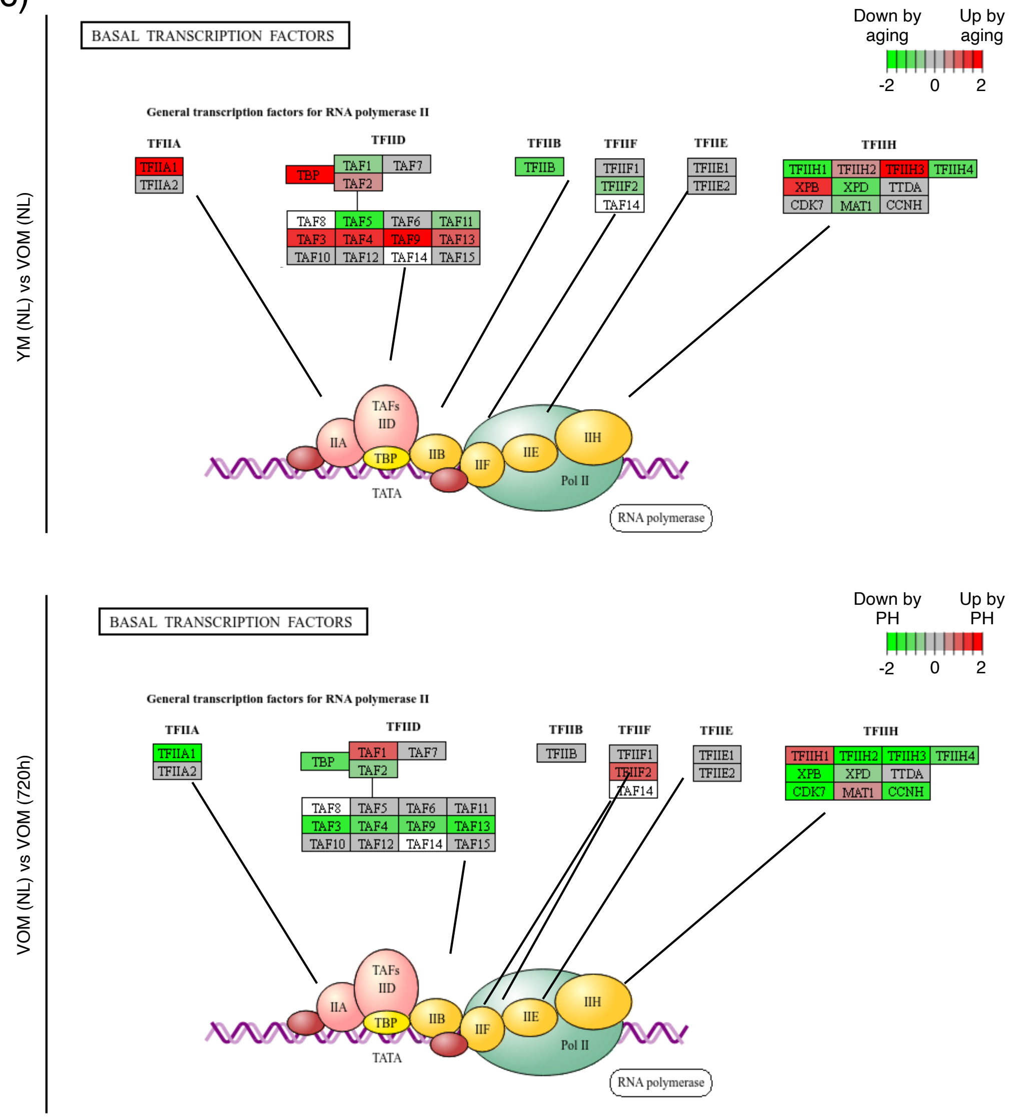

Figure S4. Gene expression changes represented on KEGG pathway maps.

(**a**-**c**) KEGG pathway maps for cholesterol metabolism (**a**), steroid biosynthesis (**b**), and basal transcription factors (**c**) are shown. Relevant genes within each pathway are indicated by rectangles. Gene expression changes are visualized using a color scale where red indicates upregulation and green indicates downregulation. The upper panels in **a** and **c** and the left panel in **b** compare gene expression levels between the normal liver (NL) of YM and VOM to illustrate aging-related gene expression changes. The lower panels in **a** and **c** and the right panel in **b** compare gene expression levels between NL of VOM and its liver at 720 h after PH to illustrate gene expression changes before and after liver regeneration.

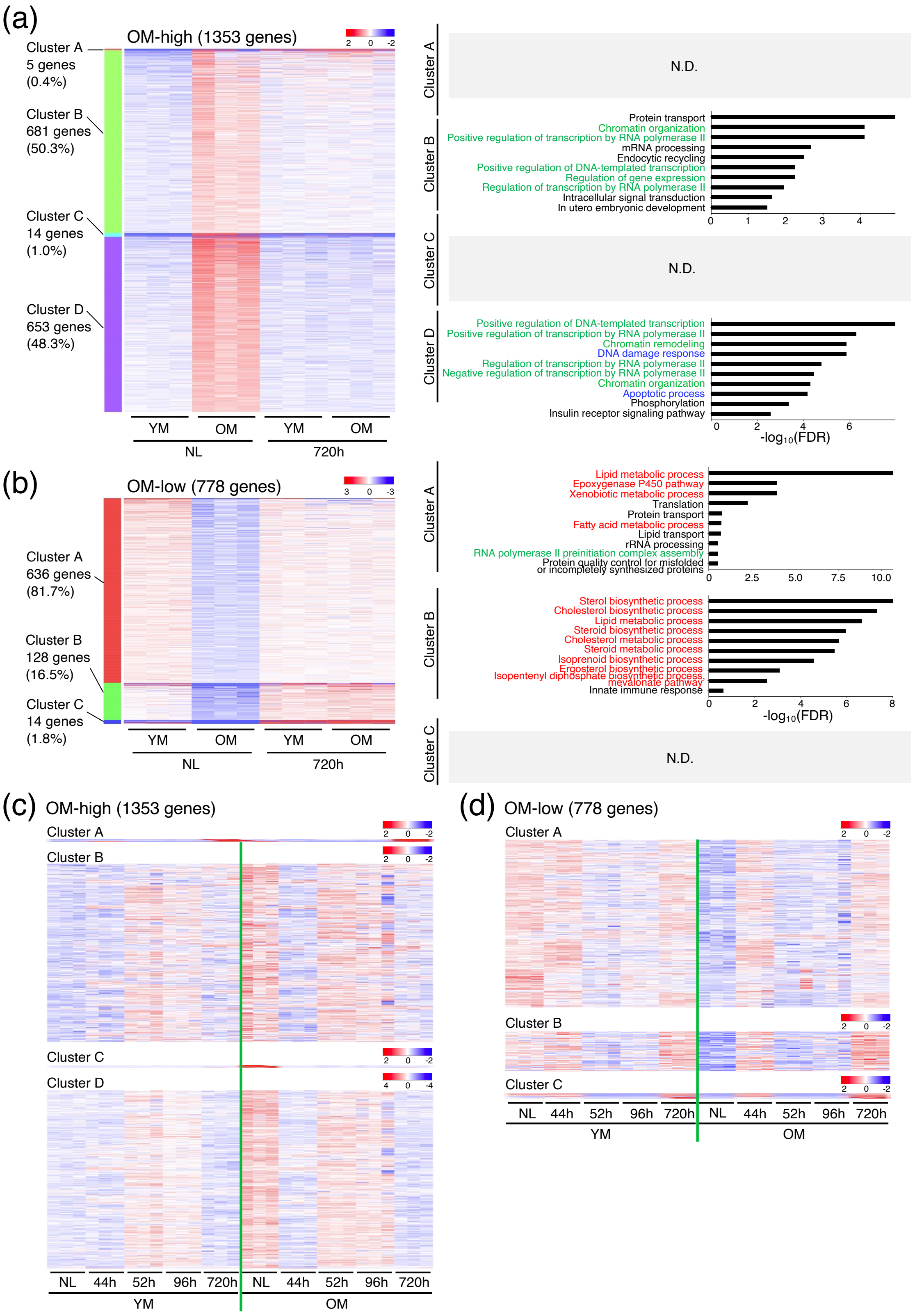

Figure S5. Changes in the expression of aging-specific genes after PH.

(**a**, **b**) Heatmap images showing changes at 720 hours (h) after PH in the expression of

aging-specific genes extracted by comparing the gene expression profiles of hepatocytes isolated from the normal liver (NL) of YM and OM. Genes with higher (**a**) and lower (**b**) expression in the NL of OM compared to YM were classified into four and three clusters, respectively. Graphs on the right depict the top-ten most significantly enriched GO terms associated with genes in each cluster, as identified by DAVID. GO terms associated with metabolic processes, apoptosis and DNA damage responses, and transcriptional regulation are highlighted in red, blue, and green, respectively. N.D., not detected. Statistical differences were determined by Fisher's exact test. (**c**, **d**) Heatmap images showing sequential expression changes of aging-specific genes classified into four (**c**) or three (**d**) clusters, which are expressed at higher or lower levels, respectively, in hepatocytes isolated from the NL of OM than in those isolated from the NL of YM.

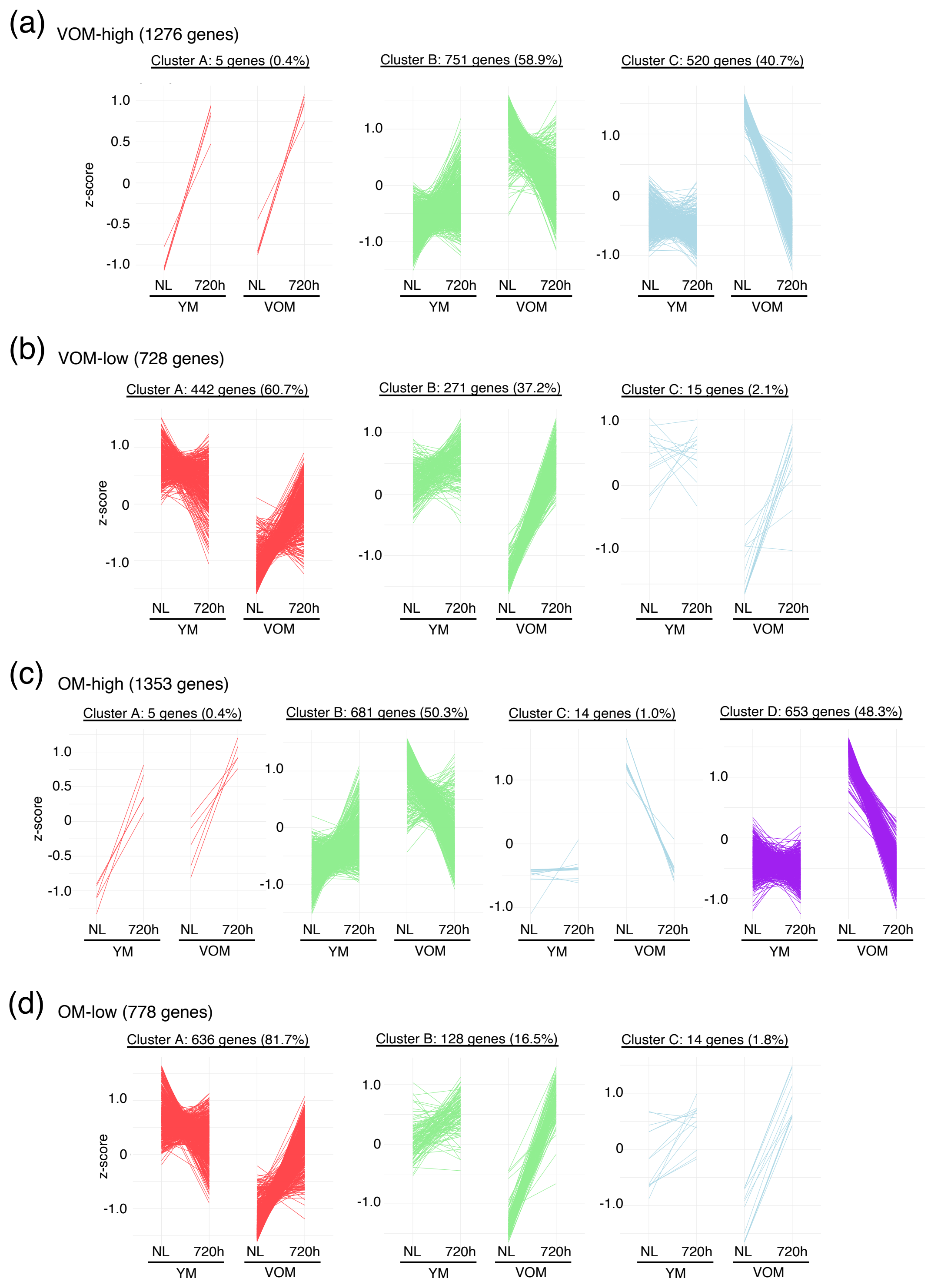

Figure S6. Classification of aging-specific genes exhibiting different patterns of expression changes in liver regeneration.

(**a-d**) Ladder plots showing changes in the expression levels of genes in each cluster shown in Figure 3 (**a**, **b**) and Figure S5 (**a**, **b**).

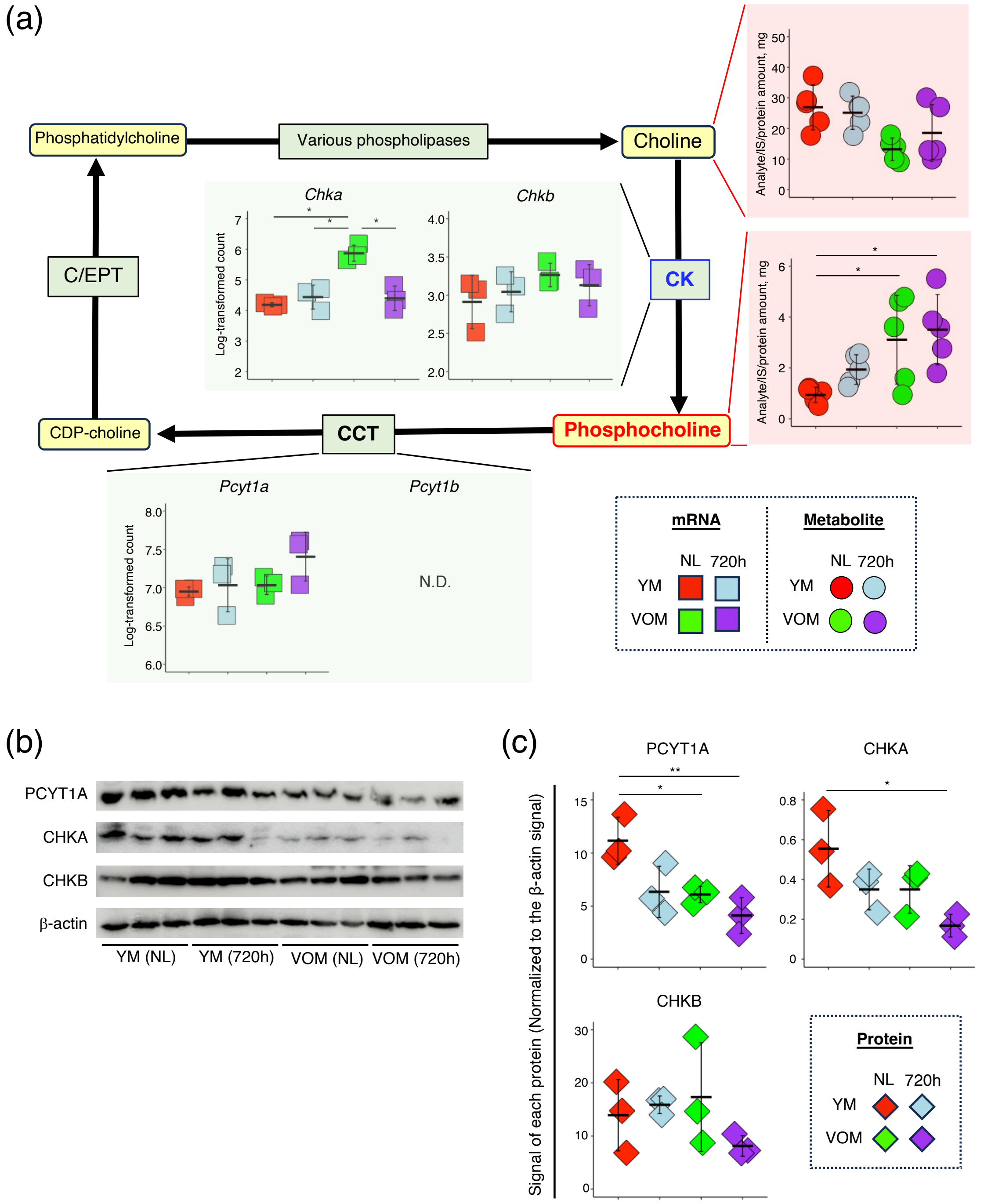

Figure S7. Expression of enzymes and levels of metabolites associated with the Kennedy pathway for phosphatidylcholine biosynthesis.

(**a**) The Kennedy pathway, which is involved in the synthesis of phosphatidylcholine, is shown. Metabolomic analysis revealed the amounts of choline and phosphocholine in the normal livers (NL) of YM and VOM, as well as in their livers at 720 h after PH (light red

graphs). Also, transcriptomic analysis revealed the gene expression levels of choline kinase (CK) isoforms *Chka* and *Chkb*, and choline-phosphate cytidylyltransferase (CCT) isoforms *Pcyt1a* and *Pcyt1b*, in the same liver samples (light green graphs). N.D., not detected. Mean values (horizontal bars) and standard deviations (vertical bars) are shown in each graph. The asterisks indicate the DEGs and DMs detected by DESeq2 and MetaboAnalyst 6.0, respectively. (**b**) Western blotting data showing the expression levels of PCYT1A, CHKA, CHKB, and β-actin in NL of YM and VOM and their livers at 720 h after PH. All proteins were analyzed with three biological replicates. (**c**) The signals obtained from western blotting were normalized to the corresponding β-actin signals in each sample. Statistical analysis was performed using one-way ANOVA for multiple comparisons, followed by Tukey's honestly significant difference test as a post-hoc analysis. $*p < 0.05$; $**p < 0.01$.
